# A Hybrid Genetic-Hierarchical Algorithm for the Quadratic Assignment Problem

**DOI:** 10.3390/e23010108

**Published:** 2021-01-14

**Authors:** Alfonsas Misevičius, Dovilė Verenė

**Affiliations:** Department of Multimedia Engineering, Kaunas University of Technology, Studentu st. 50-400/416a, LT-51368 Kaunas, Lithuania; dovile.kuznecovaite@ktu.lt

**Keywords:** combinatorial optimization, hybrid heuristic algorithms, hierarchical heuristic algorithms, genetic algorithms, tabu search, quadratic assignment problem

## Abstract

In this paper, we present a hybrid genetic-hierarchical algorithm for the solution of the quadratic assignment problem. The main distinguishing aspect of the proposed algorithm is that this is an innovative hybrid genetic algorithm with the original, hierarchical architecture. In particular, the genetic algorithm is combined with the so-called hierarchical (self-similar) iterated tabu search algorithm, which serves as a powerful local optimizer (local improvement algorithm) of the offspring solutions produced by the crossover operator of the genetic algorithm. The results of the conducted computational experiments demonstrate the promising performance and competitiveness of the proposed algorithm.

## 1. Introduction

The quadratic assignment problem (QAP) [1,2,3,4,5,6] is mathematically formulated as follows: Given two non-negative integer matrices $A={\left(a_{ij}\right)}_{n\times n}$, $B={\left(b_{kl}\right)}_{n\times n}$, and the set $\Pi_{n}$ of all possible permutations of the integers from 1 to n, find a permutation $p=\left(p\left(1\right),p\left(2\right),\ldots ,p\left(n\right)\right)\in \Pi_{n}$ that minimizes the following objective function:

(1) $$z\left(p\right)=\overset{n}{\underset{i=1}{\sum }}\overset{n}{\underset{j=1}{\sum }}a_{ij}b_{p\left(i\right)p\left(j\right)}$$

One of the examples of the applications of the quadratic assignment problem is the placement of electronic components on printed circuit boards [7,8]. In this context, the entries of the matrix A are associated with the numbers of the electrical connections between the pairs of components. The entries of the matrix B correspond to the distances between the fixed positions on the board. The permutation p=(p(1), p(2), …, p(n)) can be interpreted as a separate configuration for the arrangement of components in the positions. The element p(i) in this case indicates the number of the position to which the *i*-th component is assigned. In this way, z (or more precisely, z/2) can be understood as the total (weighted) length of the connections between the components, when all n components are placed into the corresponding n positions.

The other important areas of applications of the QAP are as follows: assigning runners in relay teams [9], clustering [10], computer/telephone keyboard design [11,12,13], planning of airport terminals [14], facility location [15], formation of chemical molecular compounds [16], formation of grey patterns [17], index assignment [18], microarray layout [19], numerical analysis [20], office assignment and planning of buildings [21,22], seed orchard design [23], turbine balancing [24], website structure design [25]. More examples of the practical applications of the QAP can be found in [4,26].

The quadratic assignment problem is also a complicated theoretical-mathematical problem. It is proved that the QAP belongs to the class of the NP-hard optimization problems [27]. The QAP can be solved exactly if the problem size (n) is quite small (n<30) [28,29,30,31,32,33,34,35]; although some special case QAP examples of larger sizes (n=36 [36], n=64 [10]) have also been exactly solved. For this reason, heuristic optimization algorithms are widely used. Although these algorithms do not guarantee the optimality of the obtained solutions, they allow for a sufficiently high quality (near-optimal) solutions within a reasonable computation time [37].

Classical single-solution local search (LS) and related algorithms were intensively used for the QAP in the early period of application of heuristic algorithms (1960–1980) [38,39,40,41]. Later, improved local search algorithms have been employed [42,43,44,45]. The so-called breakout local search has been empirically proven to be quite efficient [46,47].

Simulated annealing (SA)-based algorithms usually provide better quality results, compared to pure, deterministic local search algorithms. This applies to both the early variants of SA algorithms [48,49,50] and improved SA algorithm modifications [51,52,53].

Even more performance was achieved by adopting the tabu search (TS) methodology-based algorithms. The fast-running tabu search algorithm developed by Taillard [54] in the early 1990s is still considered as one of the most successful single-solution-based heuristic algorithms for the QAP. Since then, a number of improved variations of TS algorithms have been proposed: reactive tabu search [55], concentric tabu search [56], enhanced tabu search [57], tabu search with hardware acceleration [58], self-controlling tabu search [59], repeated iterated tabu search [60,61], parallel tabu search [62], and other variants [58,63]. The performance of LS and TS algorithms can be increased by extending these algorithms to their ameliorated “siblings”, namely, the iterated LS (ILS) [64,65] and iterated TS (ITS) algorithms [66]. Iterated search algorithms have some similarities with multistart methods [63,67,68,69] as well as greedy adaptive search procedures (GRASPs) [70].

Population-based/evolutionary algorithms constitute another important class of efficient heuristic algorithms for the QAP. The advantage of this class of algorithms is that these algorithms operate with the sets of solutions instead of single solutions and this property is of prime importance when it comes to the solution of the QAP and related problems. In particular, it is found that, namely, the genetic algorithms (GA) seem to be very likely among the most powerful heuristic algorithms for solving the QAP, among them: greedy genetic algorithm [71], genetic-local search algorithm [72,73,74], genetic algorithm using cohesive crossover [75], improved genetic algorithm [76], parallel genetic algorithm [77], memetic algorithm [78], genetic algorithm on graphics processing units [79], quantum genetic algorithm [80], and other GA modifications [81,82,83,84,85,86,87,88,89]. Note that the population-based algorithms are usually hybridized with the single-solution-based algorithms (local search, tabu search, iterated local/tabu search, GRASP). Overall, a significant part of the algorithms for the QAP are, in essence, hybrid algorithms [71,73,75,76,78,79,82,83,88,90,91,92,93,94,95,96,97,98,99,100,101,102,103,104]. It is the hybrid genetic-iterated tabu and genetic-breakout local search algorithms [76,78] that allowed to achieve the most promising results.

Swarm intelligence algorithms simulate collective intelligent behaviour of physical/biological entities (agents) (like particles (particle swarm optimization algorithms [105,106]), ants (ant colony optimization algorithms [107]), bees (artificial bee colony algorithms [108,109]). Finally, the algorithms inspired from real-world phenomena (including those using metaphors) are also applicable to the QAP [90,96,98,110,111,112,113,114,115,116,117,118]. For more extensive surveys and literature reviews on the QAP, the reader is referred to [4,119].

The main contribution of this article is that it presents an innovative hierarchicity-based genetic algorithm which is hybridized with a multi-level hierarchical iterated tabu search (HITS) algorithm serving as a powerful optimizer of the offspring solutions. The basic idea of HITS is, in turn, based on the multiple (re)use of the iterated tabu search (ITS) and, simultaneously, moving through many different locally optimal solutions. The other important novelty is that the original crossover and mutation operators are introduced. The crossover operator distinguishes for its universality and, at the same time, versatility and flexibility; while the mutation operation is integrated within the HITS algorithm and is based on combined deterministic and probabilistic (controlled random) moves between solutions during the tabu search process. Also, we have employed the modified population replacement rule. Finally, we have incorporated the population restart mechanism to avoid search stagnation. All these new features have led to the development of high-performance genetic algorithm with excellent results.

The remainder of the paper is structured as follows: In Section 2, some basic definitions are given. Then, in Section 3, the detailed description of the genetic-hierarchical algorithm and its constituent parts is provided. In Section 4, the results of the computational experiments with the proposed algorithm are presented. The paper is completed with concluding remarks.

## 2. Basic Definitions

At the beginning, we provide some preliminary (basic) formal definitions.

Suppose that p(u) (u=1,…, n) and p(v) (v=1,…, n, u≠v) are two elements of the permutation p. Then $p^{u,v}$ is defined in the following way:

(2) $$p^{u,v}\left(i\right)=\{\begin{array}{ll} p\left(i\right), & i\neq u,v\\ p\left(u\right), & i=v\\ p\left(v\right), & i=u \end{array};\;i=1,\ldots \;,n.$$

This means that the permutation $p^{u,v}$ is obtained from the permutation p by interchanging exactly the elements p(u) and p(v) in the permutation p. The formal operator (move, or transition operator) ϕ(p,u,v): $\Pi_{n}\times N\times N\to \Pi_{n}$ swaps the *u*th and *v*th elements in the given permutation such that $p^{u,v}=\phi \left(p,u,v\right)$. Note that the following equations hold: $p^{u,u}=p$, $p^{u,v}=p^{v,u}$, ${\left(p^{u,v}\right)}^{u,v}=p$.

The difference in the objective function (z) values when the permutation elements p(u) and p(v) are interchanged is calculated according to the following formula:

(3) $$\Delta \left(p^{u,v},\;p\right)=z(p^{u,v})-z\left(p\right)=\left(a_{uu}-a_{vv}\right)\left(b_{p\left(v\right)p\left(v\right)}-b_{p\left(u\right)p\left(u\right)}\right)+\;\left(a_{uv}-a_{vu}\right)\left(b_{p\left(v\right)p\left(u\right)}-b_{p\left(u\right)p\left(v\right)}\right)+\;\overset{n}{\underset{k=1,k\neq u,v}{\sum }}\left[\left(a_{uk}-a_{vk}\right)\left(b_{p\left(v\right)p\left(k\right)}-b_{p\left(u\right)p\left(k\right)}\right)+\left(a_{ku}-a_{kv}\right)\left(b_{p\left(k\right)p\left(v\right)}-b_{p\left(k\right)p\left(u\right)}\right)\right]$$

The difference in the objective function values can be calculated more faster—under condition that the previously calculated differences ($\Delta \left(p^{i,j},\;p\right)$ (i, j=1,…,n)) are memorized (stored in a matrix Ξ). The difference value is calculated using O(1) operations [54,120]:

(4) $$\Delta \left(p^{u,v},\;p\right)=z(p^{u,v})-z\left(p\right)=z\left(p\right)+\Xi \left(u,v\right).$$

After the interchange of the elements p(u) and p(v) has been performed, new differences $\Xi^{\prime }\left(i,j\right)$ (i, j=1,…,n, i≠u, i≠v, j≠u, j≠v) are calculated according this equation:

(5) $$\Xi^{\prime }\left(i,j\right)=\Xi \left(i,j\right)+\;\left(a_{iu}-a_{iv}+a_{jv}-a_{ju}\right)\left(b_{p\left(i\right)p\left(u\right)}-b_{p\left(i\right)p\left(v\right)}+b_{p\left(j\right)p\left(v\right)}-b_{p\left(j\right)p\left(u\right)}\right)+\;\left(a_{ui}-a_{vi}+a_{vj}-a_{uj}\right)\left(b_{p\left(u\right)p\left(i\right)}-b_{p\left(v\right)p\left(i\right)}+b_{p\left(v\right)p\left(j\right)}-b_{p\left(u\right)p\left(j\right)}\right).$$

If i=u or i=v or j=u or j=v, then the Formula (3) should be applied. So, all the differences are calculated using only $O\left(n^{2}\right)$ operations. Still, the initial calculation of the values of the matrix Ξ requires $O\left(n^{3}\right)$ operations (but only once before starting the optimization algorithm).

If the matrix A and/or matrix B are symmetric, then Formula (3) becomes simpler. Assume that the matrix B is symmetric. Then, the (asymmetric) matrix A can be transformed to symmetric matrix $A^{\prime }$. Thus, we get the following formula:

(6) $$\Delta \left(p^{u,v},\;p\right)=\sum_{k=1,\;k\neq u,v}^{n}\left({a^{\prime }}_{uk}-{a^{\prime }}_{vk}\right)\left(b_{p\left(v\right)p\left(k\right)}-b_{p\left(u\right)p\left(k\right)}\right);$$

here, ${a^{\prime }}_{uk}=a_{uk}+a_{ku}$, u=1,…,n, v=1,…,n, u≠v. Analogously, Formula (5) turns to the formula:

(7) $$\Xi^{\prime }\left(i,j\right)=\Xi \left(i,j\right)+\left({a^{\prime }}_{iu}-{a^{\prime }}_{iv}+{a^{\prime }}_{jv}-{a^{\prime }}_{ju}\right)\left(b_{p\left(i\right)p\left(u\right)}-b_{p\left(i\right)p\left(v\right)}+b_{p\left(j\right)p\left(v\right)}-b_{p\left(j\right)p\left(u\right)}\right).$$

If i=u(v) or j=u(v), Equation (6) must be applied.

In addition to this, suppose that we dispose of 3-dimensional matrices $A^{\prime \prime }={\left({a^{\prime \prime }}_{uvk}\right)}_{n\times n\times n}$ and $B^{\prime \prime }={\left({b^{\prime \prime }}_{lrt}\right)}_{n\times n\times n}$. Also, let ${a^{\prime \prime }}_{uvk}={a^{\prime }}_{uk}-{a^{\prime }}_{vk}$, ${b^{\prime \prime }}_{lrt}=b_{lt}-b_{rt}$, l=p(j), r=p(i), t=p(k). Then, we can apply the following formulas for calculation of the differences in the objective function values:

(8) $$\Delta \left(p^{u,v},p\right)=\sum_{k=1,k\neq u,v}^{n}{a^{\prime \prime }}_{uvk}{b^{\prime \prime }}_{p\left(v\right)k\left(u\right)p\left(k\right)};$$

(9) $$\Xi^{\prime }\left(i,j\right)=\Xi \left(i,j\right)+\left({a^{\prime \prime }}_{iju}-{a^{\prime \prime }}_{ijv}\right)\left({b^{\prime \prime }}_{p\left(j\right)p\left(i\right)p\left(v\right)}-{b^{\prime \prime }}_{p\left(j\right)p\left(i\right)p\left(u\right)}\right).$$

Using the matrices $A^{\prime \prime }$ and $B^{\prime \prime }$ allows to save up to 20% of computation (CPU) time [66]. The distance (Hamming distance) between two permutations p and $p^{\prime }$ is defined as:

(10) $$\delta \left(p,p^{\prime }\right)=\left|\left\{i:\;p\left(i\right)\neq p^{\prime }\left(i\right)\right\}\right|.$$

The following equations hold: δ(p, p)=0, $\delta \left(p,\;p^{\prime }\right)\neq 1$, $0\leq \delta \left(p,\;p^{\prime }\right)\leq n$, $\delta \left(p,\;p^{\prime }\right)=\delta \left(p^{\prime },\;p\right)$, $\delta \left(p,\;p^{u,v}\right)=2$ for any u, v (u≠v). In the case of disposing of k different numbers $u_{1},\;u_{2},\;\ldots ,\;u_{k}$, this equation holds: $\delta \left(p,{\left({\left({\left(p^{u_{1},\;u_{2}}\right)}^{u_{2},\;u_{3}}\right)}^{\ldots }\right)}^{u_{k-1},\;u_{k}}\right)=k$.

The neighbourhood function Θ: $\Pi_{n}\to 2^{\Pi_{n}}$ assigns for each $p\in \Pi_{n}$ its neighbourhood (the set of neighbouring solutions) $\Theta \left(p\right)\subseteq \Pi_{n}$. The 2-exchange neighbourhood function $\Theta_{2}$ is defined in the following way:

(11) $$\Theta_{2}\left(p\right)=\left\{p^{\prime }:p^{\prime }\in \Pi_{n},\;\delta \left(p,\;p^{\prime }\right)=2\right\};$$

where $\delta \left(p,\;p^{\prime }\right)$ is the Hamming distance between solutions p and $p^{\prime }$. The neighbouring solution $p^{\prime }\in \Theta_{2}\left(p\right)$ can be obtained from the current solution p by using the operator ϕ(p, ⋅, ⋅). The computational complexity of exploration of the neighbourhood $\Theta_{2}$ is proportional to $O(n^{2})$.

Solution $p_{loc\_opt}\in \Pi_{n}$ is said to be locally optimal with respect to the neighbourhood Θ if for every solution $p^{\prime }$ from the neighbourhood $\Theta \left(p_{loc\_opt}\right)$ the following inequality holds: $z\left(p_{loc\_opt}\right)\leq z\left(p^{\prime }\right)$.

## 3. Hybrid Genetic-Hierarchical Algorithm for the Quadratic Assignment Problem

Our proposed genetic algorithm (for more thorough information on the genetic algorithms, we refer the reader to [121]) is based on the hybrid genetic algorithm framework, where explorative (global) search is in tandem with the exploitative (local) search. The most important feature of our genetic algorithm is that it is hybridized with the so-called hierarchical (self-similar) iterated tabu search (HITS) algorithm (see Section 3.4).

The permutation elements p(1), p(2), …, p(n) are directly linked to the individuals’ chromosomes—such that the chromosomes’ genes correspond to the single elements p(1), p(2), …, p(n) of the solution p. No encoding is needed. The fitness of the individual is associated with the corresponding objective function value of the given solution, z(p).

The following are the main essential components (parts) of our genetic-hierarchical algorithm: (1) initial population construction; (2) selection of parents for crossover procedure; (3) crossover procedure; (4) local improvement of the offspring; (5) population replacement; (6) population restart. The top-level pseudo-code of the genetic-hierarchical algorithm is presented in Algorithm 1 (Notes: (1) The subroutine GetBestMember returns the best solution of the given population. (2) The mutation process is integrated within the *k*-level hierarchical iterated tabu search algorithm. The mutation process depends on the mutation variant parameter *MutVar*.).
**Algorithm 1.** High-level pseudo-code of the genetic-hierarchical algorithm.**Genetic_Hierarchical_Algorithm Procedure;**/ input: *n*—problem size, *A*, *B*—data matrices,/     *PS*—population size, *N_gen_*—total number of generations, /    *InitPopVar*—initial population variant, *SelectVar*—parents selection variant, *CrossVar*—crossover variant,/     *MutVar*—mutation variant, *RepVar*—population replacement variant,/    *σ*—selection factor, *DT*—distance threshold, *L_idle_gen_*—idle generations limit/ output: *p*^✸^—the best found solution (final solution)**begin** / create a starting population *P* of size *PS*, depending on the initial population variant*InitPopVar*** switch** (*InitPopVar*)  **1:** create the initial population *P* by applying the algorithm           **k-Level_Hierarchical_Iterated_Tabu_Search**;  **2:** create the initial population *P* by applying a copy of           **Genetic_Hierarchical_Algorithm** using *InitPopVar* = 1 **endswitch**; *p*^✸^ = GetBestMember(*P*); / initialization of the best so far solution **for**
*i* := 1 **to**
*N_gen_*
**do begin** / main loop  sort the members of the population *P* in the ascending order of the values of the         objective function;  select parents *p*′, *p*″ ∈ *P* for reproduction (crossover), depending on the selection         variant *SelectVar* and the selection factor *σ*;  perform the crossover operator on the solution-parents *p*′, *p*″         and get the offspring *p*″′, taking into account the crossover variant *CrossVar*;  apply improvement procedure **k-Level_Hierarchical_Iterated_Tabu_Search**         to the offspring *p*″′, get the (improved) offspring *p*^✩^;  get new population *P* from the union of the existing parents’         population and the offspring *P* ∪ {*p*^✩^} (such that |*P*| = *PS*)         (the minimum distance criterion and population replacement variant *RepVar*         are taken into account);  **if**
*z*(*p*^✩^) < *z*(*p*^✸^) **then**
*p*^✸^ = *p*^✩^; / the best found solution is memorized  **if** number of idle generations exceeds the predefined limit *L_idle_gen_*
**then begin**    perform the population restart process;    **if**
*z*(GetBestMember(*P*)) < *z*(*p*^✸^) **then**
*p*^✸^ = GetBestMember(*P*)   **endif**  **endfor**;  **return**
*p*^✸^**end**.

### 3.1. Creation of Initial Population

There are two main population construction phases. In the first one, the pre-initial population is constructed and improved; in the second one, the culling of the improved population is performed. So, firstly, $PS^{\prime }=PS\times C_{1}$ members of the pre-initial population P are created using the version of the GRASP algorithm [70] implemented by the authors. PS denotes the population size, and $C_{1}$ ($C_{1}\geq 1$) is the user-defined parameter and is to regulate the size of the pre-initial population.

There are several options of the population construction in the first phase controlled by the parameter InitPopVar. If InitPopVar=1, then every generated solution is improved by the hierarchical iterated tabu search algorithm. There are few conditions. If the improved solution ($p^{✩}$) is better than the best so far found solution in the population P, then the improved solution replaces the best found solution. Otherwise, it is tested if the minimum mutual distance between the improved solution ($p^{✩}$) and the existing population members ($\underset{p\in P}{\min}\left\{\delta \left(p^{✩},\;p\right)\right\}$) is greater than or equal to the predefined distance threshold, DT. If this is the case, the improved solution is added to the population P. Otherwise, the improved solution is disregarded and simply a random solution is added instead. (Remind that the distance between solutions is calculated using Equation (10)). The distance threshold is obtained from the following equation: DT=max{2, ⌊εn⌋}, where ε denotes the distance threshold factor (0<ε≤1). This presented scheme is to ensure the high level of diversity of the population members and, at the same time, to enhance the searching ability of the genetic algorithm. To obtain better initial population, the HITS algorithm with the increased number of iterations is used during the initial population formation. This is similar to a compounded approach proposed in [122].

The second option (InitPopVar=2) is almost identical to the first one, except that the genetic algorithm itself (a de facto copy of the hybrid genetic-hierarchical algorithm) (instead of the HITS algorithm) is employed for the creation of the initial population. As an alternative option (InitPopVar=3) of the population improvement, two-level genetic-hierarchical algorithm (master-slave genetic algorithm) can be employed for the initial population improvement.

In the second phase—which is very simple—$\left(C_{1}-1\right)PS$ worst members of the pre-initial population are truncated and only PS best members survive for the subsequent generations.

### 3.2. Selection of Parents

The selection of parents is performed by using the parametrized rank-based selection rule [123]. In this case, the positions ($\kappa_{1}$, $\kappa_{2}$) of the parents within the sorted population are determined according to the following formulas: $\kappa_{1}=\lfloor {\left(\varsigma_{1}\right)}^{\sigma }\rfloor$, $\kappa_{2}=\lfloor {\left(\varsigma_{2}\right)}^{\sigma }\rfloor$, $\kappa_{1}\neq \;\kappa_{2}$, where $\varsigma_{1}$, $\varsigma_{2}$ are uniform (pseudo-)random numbers in the interval $\left[1,\;PS^{\frac{1}{\sigma }}\right]$, here PS denotes the population size, and σ is a real number from the interval [1, 2] (it is referred to as a selection factor). It is clear that the better the individual, the larger the selection probability.

### 3.3. Crossover Operators

**Figure 1:**
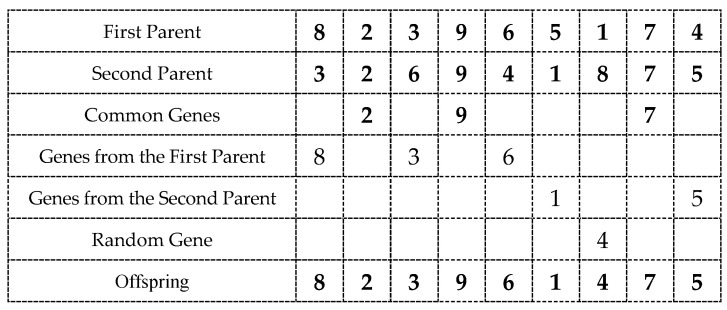
Graphical illustration of a crossover.

Two-parent crossover is formally defined by using operator $\Psi :\;\Pi_{n}\times \Pi_{n}\to \Pi_{n}$ such that:

(12) $$p{}^{\circ}=\Psi \left(p^{\prime },\;p^{\prime \prime }\right)\neq p^{\prime }\vee p{}^{\circ}=\psi \left(p^{\prime },\;p^{\prime \prime }\right)\neq p^{\prime \prime },\;p^{\prime }\neq p^{\prime \prime };$$

where $p^{\prime }$, $p^{\prime \prime }$, p° denote parental solutions, and p° is the offspring solution. (The child can coincide with one of the parents if, for example, the parents are very similar.) The crossover operator must ensure that the chromosome of the offspring necessarily inherits those genes that are common in both parent chromosomes, i.e., (also see Figure 1):

(13) $$p^{\prime }\left(i\right)=p^{\prime \prime }\left(i\right)\Rightarrow p{}^{\circ}\left(i\right)=p^{\prime }\left(i\right)\wedge p{}^{\circ}\left(i\right)=p^{\prime \prime }\left(i\right),\;i=1,2,\ldots ,n;$$

here, $p^{\prime }$, $p^{\prime \prime }$, p° refer to the parents and the offspring, respectively.

In our work, the crossover procedure takes place at every generation of the genetic-hierarchical algorithm, i.e., the crossover probability is equal to 1. Several crossover operators were implemented and examined. Short descriptions of the crossover procedures are provided below (see also [124,125]).

#### 3.3.1. One-Point Crossover—1PX

1PX is among the most popular genetic crossover operators. Very briefly, the basic idea is as follows. A single crossover point (position, or locus) is chosen in one of the two chromosomes. The position x can be determined by generating a uniformly distributed (pseudo-)random number within the interval [1, n−1] (n is the chromosome length). The offspring is obtained by copying x genes from one parent, the rest of genes are copied from the opposite parent. If there are empty loci left, they are filled in randomly; in addition, the feasibility of the offspring must be preserved.

#### 3.3.2. Two-Point Crossover—2PX

Two-point crossover works similarly to the one-point crossover, except that two crossover points $x_{1}$ and $x_{2}$ ($1\leq x_{1}<x_{2}<n$) are used.

#### 3.3.3. Uniform Crossover—UX

In this case, the common genes are copied to the offspring’s chromosome. Then, the unoccupied positions in the offspring’s chromosome are scanned form left to right and the empty loci are assigned the genes—one at a time—from one of the parents with probability $\frac{1}{2}$, i.e., $p{}^{\circ}\left(i\right)=\{\begin{array}{ll} p^{\prime }\left(i\right), & \varsigma <\frac{1}{2}\\ p^{\prime \prime }\left(i\right), & \mathrm{otherwise} \end{array}$; here, ς is a (pseudo-)random number from the interval [0, 1]. The assigned gene must be unique.

#### 3.3.4. Shuffle Crossover—SX

The shuffle crossover is obtained by randomly reordering the parents’ genes before applying the uniform crossover. The same rearrangement rule must be used for both parents. After the uniform crossover is finished, the same (initial) rearrangement rule is again applied.

#### 3.3.5. Partially-Mapped Crossover—PMX

Partially-mapped crossover can be seen as a modified variant of the *k*-point (multi-point) crossover. The basic principle relies on the so-called mapping sections (the chromosome segments between mapping points). So, at first, the segments of the chromosome of one parent are moved to the offspring’s chromosome. The same is done for the other parent. At last, the empty loci (if any) are filled in in a random way.

#### 3.3.6. Swap-Path Crossover (SPX)

##### 3.3.6.1. Swap-Path Crossover (Basic Version)—SPX1

The main distinguishing feature of SPX is that instead of transferring genes from parents to a child, the genes are, so to speak, rearranged in the chromosomes of the parents. Let $\left(p^{\prime },\;p^{\prime \prime }\right)$ be a pair of parents. Then, the process starts from an arbitrary position and the positions are scanned from left to right. The process continues until a predefined number of swaps, s (s<n), have been performed. If, in the current position, the genes are the same for both parents, then one moves to the next position; otherwise, a pairwise interchange of genes of the parents’ chromosomes is accomplished. The interchange is performed in both parents. For example, if the current position is i and $a=p^{\prime }\left(i\right)$, $b=p^{\prime \prime }\left(i\right)$, then there exists a position j such that $b=p^{\prime }\left(j\right)$, $a=p^{\prime \prime }\left(j\right)$; then, after a swap, $p^{\prime }\left(i\right)=b$, $p^{\prime \prime }\left(i\right)=a$ and $p^{\prime }\left(j\right)=a$, $p^{\prime \prime }\left(j\right)=b$. Consequently, new chromosomes, say $p^{\prime \prime \prime }$, $p^{⁗}$, are produced. In the next iteration, a pair $\left(p^{\prime \prime \prime },\;p^{⁗}\right)$ is considered, and so on.

##### 3.3.6.2. Swap-Path Crossover (Modified Version I)—SPX2

This modification is achieved when the best offspring (with respect to the fitness of the offspring) is retained in the course of gene interchanges.

##### 3.3.6.3. Swap-Path Crossover (Modified Version II)—SPX3

The essential feature this crossover procedure is that the offspring fitness is dynamically evaluated: only the gene interchanges that improve the value of the objective function are accepted.

##### 3.3.7. Cycle Crossover—CX

The cycle crossover is based on the pairwise gene interchanges. The key property of CX is the ability to produce the offspring without distortion of the genetic code; in the other words, CX enables to create the chromosome with no random (foreign) genes. The negative aspect of CX is that the offspring may genetically be very close to their predecessors.

##### 3.3.8. Cohesive Crossover—COHX

The cohesive crossover was proposed by Z. Drezner [75] to efficiently recombine individuals’ genes by taking into account the problem data, in particular, the distances between objects’ locations. From several recombinations of genes, the recombination is selected that minimizes the objective function.

##### 3.3.9. Multi-Parent Crossover—MPX

In the multi-parent crossover, several (or all) members of a population participate in creation of the offspring. More precisely, the *i*th position (locus) of the offspring’s chromosome p° is assigned the value j with the probability P(p(i)=j) (under condition that the value j has not been utilized before).

The probability that p(i)=j (P(p(i)=j)) is calculated according to the formula: $P\left(p\left(i\right)=j\right)=\frac{q_{ij}}{\sum_{j=1}^{n}q_{ij}}$; where $q_{ij}$ is an element of the matrix $Q={\left(q_{ij}\right)}_{n\times n}$; here, $q_{ij}$ denotes the number of times that the *i*th locus takes the value j in the parental chromosomes. If there exist several values ($j_{1}$, $j_{2}$, …) with the same probability, then one of them is chosen randomly.

##### 3.3.10. Universal Crossover—UNIVX

The universal crossover (UNIVX) [124] distinguishes for its versatility and the possibility of flexible usage depending on the specific needs of the user. It is somewhat similar to what is known as a simulated binary crossover [126].

Our operator is based on the use of a random mask. There are four controlling parameters: $\chi_{1}$, $\chi_{2}$, $\chi_{3}$, $\chi_{4}$. The mask length is equal to $\chi_{1}$, where $\chi_{1}$ is a (pseudo-)random number within the interval $\left[\varepsilon_{1},\;n\right]$, n is the length of the chromosome, $\varepsilon_{1}=\lfloor r\times n\rfloor$, r is the user’s parameter close to 1, for example, 0.9. The mask contains binary values 0 and 1, where 1 signals that the corresponding gene of the first parent’s chromosome must be chosen and 0 is to indicate that the second parent’s gene needs to be replicated. The degree of randomness of the mask is controlled by the parameters $\chi_{2}$, $\chi_{3}$. The parameter $\chi_{2}$ ($\chi_{2}\in \left[\varepsilon_{2},\;\varepsilon_{3}\right]$, $0<\varepsilon_{2}\leq \varepsilon_{3}<1$) dictates how many 0’s and 1’s are there in the mask: the higher the value of $\chi_{2}$, the bigger total number of 1’s, and vice versa. The juxtaposition of bits is regulated by the parameter $\chi_{3}$. The bit generation itself is performed by using a kind of “anytime” min-max sorting algorithm. That is, if the sorting algorithm is interrupted at some random moment, this results in a randomized (“quasi-sorted”) sequence of bits. The moment of interruption is defined by the number η, where $\eta =\chi_{3}w$, here $\chi_{3}$ is a (pseudo-)random real number from the interval [0, 1], and w denotes the maximum number of iterations required to fully sort all the bits. (As an example, if the bits “0000001111” are to be sorted in the descending order and the algorithm is stopped at $\chi_{3}=0.9$, then the random mask similar, for example, to “1011000100” would be generated.) Having the mask generated, the decision is made as to about what genes have to be transmitted to the offspring’s chromosome. The index of the starting locus of the transferred genes, $\chi_{4}$, is generated randomly—in such a way that $\chi_{4}\in \left[1,\;n\right]$. Eventually, the empty loci (if any) are filled in randomly.

### 3.4. Offspring Improvement

#### 3.4.1. Hierarchical Iterated Tabu Search Algorithm

**Figure 2:**
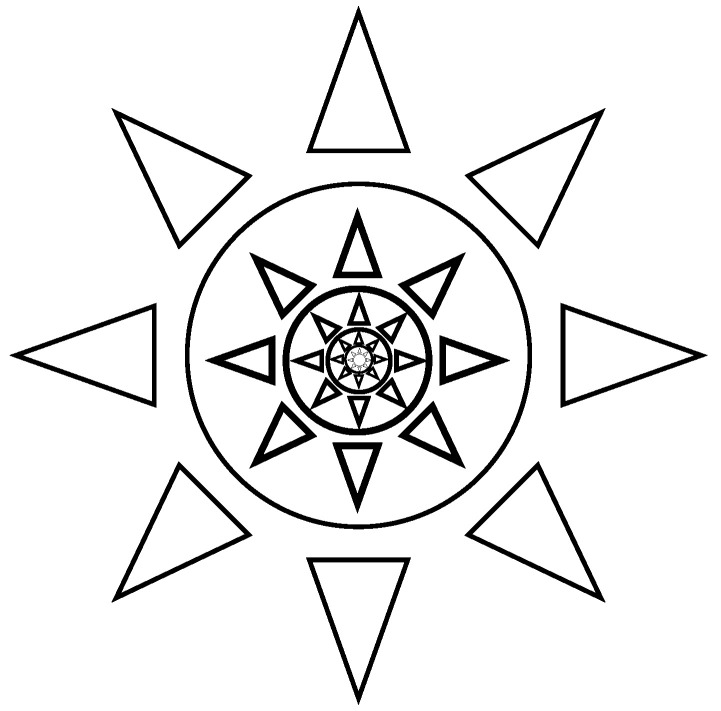
Visual conceptual interpretation of hierarchicity.

Every created offspring is improved by using the hierarchical iterated tabu search algorithm, which inspires from the philosophy of iterated local search [127] and also the spirit of self-similarity—one of the fundamental properties of nature (see Figure 2). Basically, this means that the algorithm is (almost) exactly similar to the part of itself. In the other words, the main idea is the repeated, cyclical adoption (reuse) of the iterated tabu search algorithm, that is, the iterated tabu search can be reused multiple times. This idea is not very new, and some variants of hierarchical-like algorithms have been already investigated [128,129,130,131,132,133,134].

The paradigm of the hierarchicity based (self-similar) algorithm is as follows:

(1) Set the number of levels, k ($1\leq k\leq k_{max}$).
(2) Generate an initial solution p.
(3) Apply *k*‒1-level algorithm to the solution p. Let $p^{✸}$ be the improved solution.
(4) Memorize the best found solution.
(5) Set $p^{☼}=p^{✸}$ or select a solution $p^{☼}$ from the history of solutions.
(6) Apply a perturbation procedure to the solution $p^{☼}$. Let $p^{\sim }$ be the perturbed solution.
(7) Set $p=p^{\sim }$.
(8) If the termination criterion is not satisfied, then go to Step 2; otherwise, stop the algorithm.

The *k*-level hierarchical iterated tabu search algorithm consists of three basic components: (1) invocation of the *k*–1-level hierarchical iterated tabu search algorithm to improve a given solution; (2) acceptance of the candidate (improved) solution for perturbation, i.e., mutation; (3) mutation of the accepted solution.

Perturbation (mutation) is applied to a chosen optimized solution that is selected by the defined candidate acceptance rule (see Section 3.4.3). The mutated solution serves as an input for the self-contained TS procedure. The TS procedure returns an improved solution, and so on. The overall process continues until a pre-defined number of iterations have been performed (see Algorithm 2 (Note. The iterated tabu search procedure (see Algorithm 3) is in the role of the 0-level hierarchical iterated tabu search algorithm.)). The best found solution is regarded as the resulting solution of HITS.
**Algorithm 2.** Pseudocode of the *k*-level hierarchical iterated tabu search algorithm.**k-Level_Hierarchical_Iterated_Tabu_Search procedure**;/ input: *p*—current solution/ output: *p*^✩^—the best found solution/ parameter: *Q*^〈*k*〉^—number of iterations of the *k*-level HITS algorithm**begin** *p*^✩^: = *p*; **for**
*q*^〈*k*〉^: = 1 **to**
*Q*^〈*k*〉^
**do begin**  apply **k–1-Level_Hierarchical_Iterated_Tabu_Search** to *p* and get *p*^∇^;  **if**
*z*(*p*^∇^) < *z*(*p*^✩^) **then**
*p*^✩^: = *p*^∇^; / the best found solution is memorized  **if**
*q*^〈*k*〉^ < *Q*^〈*k*〉^
**then begin**   *p*: = **Candidate_Acceptance**(*p*^∇^, *p*^✩^);   apply mutation procedure to *p*  **endif** **endfor****end**.
**Algorithm 3.** Pseudocode of the iterated tabu search algorithm.**Iterated_Tabu_Search procedure**;/ 0-level hierarchical iterated tabu search algorithm/ input: *p*—current solution/ output: *p*^〈0〉^—the best found solution/ parameter: *Q*^〈0〉^—number of iterations of the ITS algorithm**begin** *p*^〈0〉^: = *p*; **for**
*q*^〈0〉^ := 1 **to**
*Q*^〈0〉^
**do begin**  apply **Tabu_Search** to *p* and get *p*^•^;  **if**
*z*(*p*^•^) < *z*(*p*^〈0〉^) **then**
*p*^〈0〉^: = *p*^•^; / the best found solution is memorized  **if**
*q*^〈0〉^ < *Q*^〈0〉^
**then begin**   *p*: = **Candidate_Acceptance**(*p*^•^, *p*^〈0〉^);   apply mutation procedure to *p*   **endif** **endfor****end**.

The 0-level HITS algorithm is in fact simply iterated tabu search algorithm (for more details on the ITS algorithm, see [135]) (see Algorithm 3 ((Note. The tabu search procedure is in the role of the self-contained algorithm.))), which, in turn, uses a self-contained tabu search algorithm—the “kernel” tabu search procedure. It is this procedure that directly improves a given solution. This procedure is thus in the role of the search intensification mechanism, while the mutation procedure is responsible for the diversification of the search process. It can be seen that the structure of the individual hierarchical levels of the HITS algorithm is quite simple, but the overall efficacy of the resulting multi-level algorithm increases significantly, which is demonstrated by the computational experiments. Of course, the run time increases as well, but this is compensated by the higher quality of the final results.

The interesting analogy between intensification and diversification (on the one side) and the phenomenon of entropy (on the other side) can be perceived. Indeed, the intensification process can be thought of as a process of the decrease of the entropy; on the other hand, diversification could be viewed as the increase of the entropy. Actually, the similar processes are seen in the open real physical systems. An example is the process of evolution of stars, where formation (birth) of the stars (along with the planets, organic matter, etc.) can be linked to the apparent decrease of the entropy, while the death of the stars (supernovae) may be associated with the significant increase of the entropy.

The self-contained tabu search procedure (for a more detailed description of the principles of TS algorithms, the reader is referred to [136]) iteratively analyses the neighbourhood of the current solution p (in our case—$\Theta_{2}\left(p\right)$) and performs the non-prohibited move that most improves the value of the objective function. If there are no improving moves, then the one that least degrades the value of the objective function is accepted. In order to eliminate search cycles, the return to recently visited solutions is disabled for a specified period. The list of prohibitions—the tabu list T—is implemented as a two-dimensional matrix of size n×n. In this case, the entry $t_{ij}$ stores the sum of the number of the current iteration and the tabu tenure, h; in this way, this value indicates from which iteration the *i*th and *j*th elements of a given solution can be again interchanged. The value of the parameter h depends on the problem size, n, and is chosen to be equal to 0.3n. The tabu status is ignored at random moments with a very low probability α (α≤0.05). This allows to slightly increase the number of non-tabu solutions and not to limit the available search directions too much. The tabu condition is also ignored when the aspiration criterion is met, i.e., the current obtained solution is better than the best so far found solution. The resulting formal tabu and aspiration criteria are thus as follows:

$tabu\_criterion^{\left(i,j\right)}=\{\begin{array}{ll} \mathrm{TRUE}, & \left(t_{ij}\geq q\right)\;\mathrm{and}\;\left(\varsigma \geq \alpha \right)\;\mathrm{and}\;\left(HT\left(\left(z\left(p\right)+\Delta \left(p^{i,j},\;p\right)\right)\mathrm{mod}\;HashSize\right)=\mathrm{TRUE}\right)\\ \mathrm{FALSE}, & \mathrm{otherwise} \end{array}$, $aspiration\_criterion^{\left(i,j\right)}=\{\begin{array}{ll} \mathrm{TRUE}, & z\left(p\right)+\Delta \left(p^{i,j},\;p\right)<z^{•}\\ \mathrm{FALSE}, & \mathrm{otherwise} \end{array}$, where i, j are the current elements’ indices, q denotes the current iteration number, ς is a (pseudo-)random real number within the interval [0, 1], and $z^{•}$ denotes the best so far found value of the objective function. HT denotes the hash table, which is simply a one-dimensional array, and HashSize is the capacity of the hash table.

In addition, our tabu search procedure uses a so-called secondary memory Γ [137] to avoid stagnation manifestations during the search process. The purpose of this memory is to gather high-quality solutions, which although are rated as very good, but are not chosen. In particular, the secondary memory includes solutions left “second” after the exploration of the neighbourhood $\Theta_{2}$. So, if the best found solution does not change more than ⌊βτ⌋ iterations, then the tabu list is cleared and the search is restarted from one of the “second” solutions in the secondary memory Γ (here, τ denotes the number of iterations of the TS procedure, and β is a so-called idle iterations limit factor such that 1≤⌊βτ⌋≤τ). The TS procedure is completed as soon as the total number of iterations, τ, has been performed.

The time complexity of the TS algorithm is proportional to $O\left(n^{2}\right)$ for the reason that the exploration of the neighbourhood $\Theta_{2}$ requires $\frac{n\left(n-1\right)}{2}$ operations and also one needs to recalculate the differences of the objective function after each transition from a given solution to the new one.

The pseudo-code of the tabu search algorithm is shown in Algorithm 4 (Notes. (1) The immediate if function iif(x, $y_{1}$, $y_{2}$) returns $y_{1}$ if x=TRUE, otherwise it returns $y_{2}$. (2) The function random() returns a pseudo-random number uniformly distributed in [0, 1]. (3) The function random($x_{1}$, $x_{2}$) returns a pseudo-random number in $\left[x_{1},\;x_{2}\right]$. (4) The values of the matrix Ξ are recalculated according to the Formula (9). (5) β denotes a random access parameter (we used β=0.8).).
**Algorithm 4.** Pseudo-code of the tabu search algorithm.**Tabu_Search procedure**;/input: *n*—problem size,/     *p*—current solution, Ξ—difference matrix/output: *p*^•^—the best found solution (along with the corresponding difference matrix)/parameters: *τ*—total number of tabu search iterations, *h*—tabu tenure, *α*—randomization coefficient,/          *L_idle_iter_*—idle iterations limit**begin** clear tabu list *TabuList* and hash table *HashTable*; *p*^•^: = *p*; *k*: = 1; *k*′: = 1; *secondary_memory_index*: = 0; *improved*: = FALSE; **while** (*k* ≤ *τ*) **or** (*improved* = TRUE) **then begin** / main cycle  Δ′*_min_*: = ∞; Δ″*_min_*: = ∞; *u*′: = 1; *v*′: = 1;  **for**
*i*: = 1 **to**
*n* − 1 **do**   **for**
*j*: = *i* + 1 **to**
*n*
**do begin** / *n*(*n* − 1)/2 neighbours of *p* are scanned    Δ: = Ξ(*i*, *j*);    *forbidden*: = **iif**(((*TabuList*(*i*, *j*) ≥ *k*) **and** (*HashTable*((*z*(*p*) + Δ) **mod**
*HashSize*) = TRUE) **and**           (random() ≥ *α*)), TRUE, FALSE);    *aspired* := **iif**(*z*(*p*) + Δ < *z*(*p*^•^), TRUE, FALSE);    **if** ((Δ < Δ′*_min_*) **and** (*forbidden* = FALSE)) **or** (*aspired* = TRUE) **then begin**     **if** Δ < Δ′*_min_*
**then begin** Δ″*_min_*: = Δ′*_min_*; *u*″: = *u*′; *v*″: = *v*′; Δ′*_min_*: = Δ; *u*′: = *i*; *v*′: = *j*; **endif**     **else if** Δ < Δ″*_min_*
**then begin** Δ″*_min_*: = Δ; *u*″: = *i*; *v*″: = *j*; **endif**    **endif**   **endfor**;  **if** Δ″*_min_* < ∞ **then begin** / archiving second solution, Ξ, *u*″, *v*″   *secondary_memory_index*: = *secondary_memory_index* + 1; Γ(*secondary_memory_index*) ← p, Ξ, u″, v″  **endif**;  **if** Δ′*_min_* < ∞ then begin / replacement of the current solution and recalculation of the values of Ξ   $p:\;=\;\phi \left(p,\;u^{\prime },\;v^{\prime }\right)$;   recalculate the values of the matrix Ξ;   **if**
*z*(*p*) < *z*(*p*^•^) **then begin**
*p*^•^: = *p*; *k*′: = *k*
**endif**; / the best so far solution is memorized   *TabuList*(*u*′, *v*′): = *k* + *h*; / the elements *p*(*u*′), *p*(*v*′) become tabu   *HashTable*((*z*(*p*) + Δ) **mod**
*HashSize*): = TRUE  **endif**;  *improved*: = **iif**(Δ′*_min_* < 0, TRUE, FALSE);  **if** (*improved* = FALSE) **and** (*k* − *k*′ > *L_idle_iter_*) **and** (*k* < *τ* − *L_idle_iter_*) **then begin**   / retrieving solution from the secondary memory   *random_access_index*: = random(β × *secondary_memory_index*, *secondary_memory_index*);   *p*, Ξ, *u*″, *v*″ ← Γ(*random_access_index*);   $p:\;=\;\phi \left(p,\;u^{\prime \prime },\;v^{\prime \prime }\right)$;   recalculate the values of the matrix Ξ;   clear tabu list *TabuList*;   *TabuList*(*u*″, *v*″): = *k* + *h*; / the elements *p*(*u*″), *p*(*v*″) become tabu   *k*′: = *k*  **endif**;  *k*: = *k* + 1 **endwhile****end**.

#### 3.4.2. Mutation

Each solution found by the tabu search algorithm is subject to perturbation in the mutation procedure. Remind that formally the mutation process can be defined by the use of the operator φ: $\Pi_{n}\to \Pi_{n}$. Thus, if $p^{\sim }=\varphi \left(p\right)$, then $p^{\sim }\in \Pi_{n}$, $p^{\sim }\neq p$. More formalized operator can be described as follows: φ(p, ξ): $\Pi_{n}\times N\to \Pi_{n}$, which transforms the current solution p to the new solution $p^{\sim }$ such that $\delta \left(p,\;p^{\sim }\right)=\delta \left(p,\;\varphi \left(p\right)\right)=\xi$. In this way, $\frac{100\xi }{n}$ per cent elements of the solution are affected. The parameter ξ (2≤ξ≤n) regulates the strength of mutation and is referred to as a mutation rate. (In our algorithm, ξ=⌊0.2n⌋.) It is clear that for any p, $p^{\sim }$ (such that $p\neq p^{\sim }$, $\delta \left(p,\;p^{\sim }\right)=\xi$) there always exists a sequence of distinct integers $u_{1},u_{2},\ldots ,\;u_{\xi }$ such that $p^{\sim }={\left({\left({\left(p^{u_{1},\;u_{2}}\right)}^{u_{2},\;u_{3}}\right)}^{\ldots }\right)}^{u_{\xi -1},\;u_{\xi }}$.

Choosing the right value of the mutation rate, ξ, plays a very important role in the mutation procedure and the HITS algorithm and, at the same time, the whole genetic algorithm. A proper compromise between two extreme cases must be achieved: (1) the value of ξ is (very) low (close to 0); (2) the value of ξ is (very) high (close to n). In the first case, the mutation would not guarantee that the obtained mutated solution is “far” away enough from a given solution, which would lead to cycling search trajectories. In the second case, useful accumulated information would be lost and the algorithm would become very similar to a crude random multi-start method.

It should be stressed that the mutation processes are quite different from the crossover procedures. Mutation processes are by their nature purely random processes. Whilst crossover procedures only recombine the genetic code contained in the parents, the mutation processes generate, in essence, new information that hadn’t existed in predecessors earlier. It is the mutation process that implicitly is a true creative process and potentially produces a real novelty. In our work, twelve different mutation procedures and their modifications were proposed and tested.

##### 3.4.2.1. Mutation Based on Random Pairwise Interchanges (M1)

**Figure 3:**
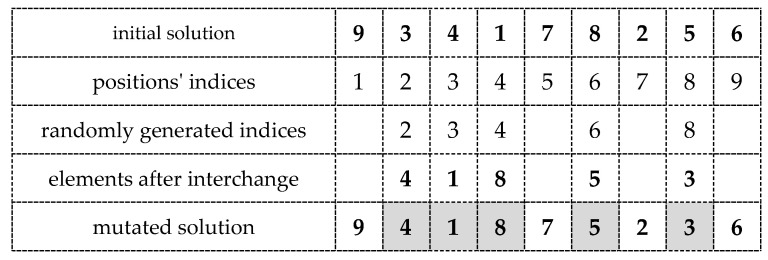
Illustration of the mutation procedure (n=9, ξ=5) (The mutation process steps are as follows: (1) element 3 is interchanged with element 4; (2) element 3 (in position 3) is interchanged with element 1; (3) element 3 (in position 4) is interchanged with element 8; (4) element 3 (in position 6) is interchanged with element 5 (element 3 is eventually in position 8)).

In the beginning, the sequence r=(r(1), r(2), …, r(ξ)) of random integers r(i)∈{1, …., n} is generated. Then, we start with the pair (r(1), r(2)), and the elements p(r(1)), p(r(2)) are interchanged. Then, we exchange the elements p(r(2)), p(r(3)), and so on. This is repeated ξ−1 times, where ξ is the value of the mutation rate defined by the algorithm’s user. The result of the mutation procedure is thus the solution $p^{\sim }$ satisfying the conditions: $p^{\sim }\in \Pi_{n}$, $\delta \left(p,\;p^{\sim }\right)=\xi$ (see Figure 3).

On the basis of the random pairwise interchanges, other modified mutation procedures can be developed [138].

##### 3.4.2.2. Random Pairwise Interchanges Using Random Key (M2)

In this case, the mutation process consists of two basic steps: (1) random pairwise interchanges; (2) shuffling the interchanged elements using a random key. A random key, rk, is a list of random indices of size ξ—rk(1), rk(2), …, rk(ξ). The random key values simply determine which elements are again interchanged. The intention is to get a more “deeply” mutated solution and avoid returning to previously visited solutions.

##### 3.4.2.3. Mutation Using Opposite Values (M3)

In this mutation procedure, the position’s index, let’s say k, is randomly determined. Then, the element e=p(k) is replaced by the following opposite value: $o=\left(\left(p\left(k\right)+\frac{n}{2}-1\right)\;\mathrm{mod}\;n\right)+1$, where mod denotes the modulo operation. After this replacement, the solution element that was previously equal to o must also be changed. After both changes, p(k) becomes equal to o, p(l)—equal to e; l indicates the element which was equal to o. The process is repeated $\frac{\xi }{2}$ times, where ξ is the muation rate.

##### 3.4.2.4. Distance-Based Mutation (M4)

In this procedure, the indices of the pairs of elements to be interchanged are generated in such a way that the “distance” (d) between those indices is as large as possible. The following formula for generating the indices $k_{1},\;k_{2},\;\ldots ,\;k_{\xi }$ is used: $k_{l}=\lfloor \left(\left(dq_{l}+\varsigma -1\right)\;\mathrm{mod}\;n\right)+1\rfloor$, here $d=\frac{n}{\xi }$, ς—(pseudo)random real number from the interval [0, 1], $q_{l}=\left(q_{l-1}\;\mathrm{mod}\;n\right)+1$, l=1,2,…, ξ; the initial value $q_{0}$ is a random integer from the interval [1,n].

##### 3.4.2.5. Modified Random Pairwise Interchanges—Variant I (M5)

This is similar to the random pairwise interchanges. The sequence of random real-coded values from the interval [0,  1] is generated. The generated numbers along with their corresponding indices—known as smallest positive values—are sorted in the ascending order. These values, in particular, determine the elements to be interchanged.

##### 3.4.2.6. Modified Random Pairwise Interchanges—Variant II (M6)

The list of random indices is obtained by directly generating random integers from the interval [1, n]. The integers may duplicate each other. To avoid duplications, the integers are sorted according to the ascending order. Indices corresponding to the sorted numbers indicate the elements that are to be interchanged.

##### 3.4.2.7. Combined Mutation (M7)

This mutation procedure consists of two combined mutation procedures. Initially, the list of indices of the pairs of elements to be interchanged is constructed (see Section 3.4.2.6). The selected elements are then changed using opposite values (see Section 3.4.2.3).

##### 3.4.2.8. Greedy Adaptive Search Based Mutation (M8)

The basic principle of this mutation procedure is that the solution is disintegrated in some way, and then reconstructed. The mutation process consists of two steps: (1) disintegration of the solution, which is random; (2) reconstruction of the solution, which is greedy-deterministic. In the first step, ξ elements are disregarded. In the second step, a greedy constructive algorithm is applied, which tries to find the best possible solution out of ξ! available options. The value of ξ in this case should be quite small to prevent large increase in the run time of the mutation procedure. This mutation procedure (and also other procedures described below) are no longer problem-independent as the problem domain-specific knowledge is taken into account.

##### 3.4.2.9. Greedy Randomized Adaptive Search Based Mutation (M9)

This mutation procedure resembles the one described above. The difference is that a greedy randomized adaptive search procedure (GRASP) [70] is used in the partial solution reconstruction phase to obtain an improved solution.

##### 3.4.2.10. Randomized Local Search Based Mutation—Variant I (M10)

In this case, quick procedure based on random pairwise interchanges is initially performed (see Section 3.4.2.1). Then, a set of randomly selected elements is formed. A local search is then performed using the constructed set, i.e., only transitions between solutions that improve the value of the objective function are accepted.

##### 3.4.2.11. Randomized Local Search Based Mutation—Variant II (M11)

This mutation variant is similar to the previous randomized local search variant, except that the randomly constructed neighbourhood is fully explored in a systematic way. Again, only improving transitions between solutions are accepted.

##### 3.4.2.12. Randomized Tabu Search Based Mutation (M12)

Let $p^{\left(1\right)}=\underset{i=1,\;\ldots ,\;n-1,\;j=i+1,\;\ldots ,\;n,\;move\_acceptance\_criterion^{\left(i,j\right)}=\mathrm{TRUE}}{\mathrm{argmin}}\left\{z\left(p^{i,j}\right)\right\}$ and

$p^{\left(2\right)}=\underset{i=1,\;\ldots ,\;n-1,\;j=i+1,\;\ldots ,\;n,\;move\_acceptance\_criterion^{\left(i,j\right)}=\mathrm{TRUE}}{\mathrm{argmin}}\left\{z\left(p^{i,j}\right)|p^{i,j}\neq p^{\left(1\right)}\right\},$ where:$move\_acceptance\_criterion^{\left(i,j\right)}=\{\begin{array}{ll} \mathrm{TRUE}, & \left(tabu\_criterion^{\left(i,j\right)}=\mathrm{FALSE}\right)\;\mathrm{or}\;\left(aspiration\_criterion^{\left(i,j\right)}=\mathrm{TRUE}\right)\\ \mathrm{FALSE}, & \mathrm{otherwise} \end{array}$, $tabu\_criterion^{\left(i,j\right)}=\{\begin{array}{ll} \mathrm{TRUE}, & \left(t_{ij}\geq q\right)\;\mathrm{and}\;\left(\varsigma \geq \alpha \right)\\ \mathrm{FALSE}, & \mathrm{otherwise} \end{array}$,

$aspiration\_criterion^{\left(i,j\right)}=\{\begin{array}{ll} \mathrm{TRUE}, & z\left(p\right)+\Delta \left(p^{i,j},\;p\right)<z^{•}\\ \mathrm{FALSE}, & \mathrm{otherwise} \end{array}$, q denotes the current iteration number, ς is a (pseudo-)random number within the interval [0, 1], α denotes the randomization coefficient, $z^{•}$ denotes the best so far value of the objective function. Then, in the randomized tabu search procedure, the best achieved solution (“winner solution”) $p^{\left(1\right)}$ is accepted with the probability γ, meanwhile the second solution $p^{\left(2\right)}$ is chosen with the probability 1−γ (note that, in the case of γ=1, we get the standard (deterministic) tabu search.) In our algorithm, we use γ=0.2. So, the central idea of the randomized tabu search is just this quasi-random mixing between the “winner solution” and “next to the winner solution” in the course of the tabu search process. Based on the extensive experimentation, we found out that this type of mutation is the most promising mutation procedure among all the procedures examined. The explanation would be that this type of mutation rather is more gentle, moderate and controlled than the other mutation procedures.

In the end, note that the computational complexity of all our mutation procedures is proportional to $O\left(\xi n^{2}\right)$. This is due to the fact that our mutation procedures recalculate the differences of the objective function (i.e., the values of the matrix Ξ) approximately ξ times (see Algorithm 5 (Note. The values of the matrix Ξ are recalculated using Equation (9).)). So, the smaller the value of ξ, the faster is the mutation procedure. Also, note that the difference matrix Ξ is (permanently) stored in a RAM (operating memory), so there is no need to calculate the differences of the objective function from scratch.

**Algorithm 5.** Pseudo-code of the procedure for recalculation of the differences of the objective function.**Recalculation_of_the_Differences_of_the_Objective_Function procedure**;/ input: *ξ*—mutation rate, *p*—initial permutation before mutation, *r*—random index array, Ξ—current differences/ output: Ξ—new differences
**begin**
 **for**
*l*: = 1 **to***ξ* − 1 **do begin**  u: = r(l); v: = r(l + 1); p: = ϕ(p, u, v);  recalculate values of the matrix Ξ **endfor****end**.

#### 3.4.3. Candidate Acceptance

Regarding the candidate solution acceptance rule, we always choose the most recently (newly) found improved solution (the latest result of the HITS (or TS) algorithm) instead of the overall best found solution. Such an approach is thought to allow to explore potentially larger regions of the solution space.

### 3.5. Population Replacement

For the population replacement, a modified rule is used to respect not only the quality of the solutions, but also the difference (distance) between solutions.

We have, in particular, implemented an extended variant of the well-known “μ+λ“ update rule [139]. The new advanced replacement rule is denoted as “μ+λ,ε”. (This rule is also used for the initial population construction (see Section 3.1).) We remind that if the minimum mutual distance between population members and the new obtained offspring is less than the distance threshold, DT, then the offspring is omitted. The only exception is the case where the offspring appears better than the best population member. Otherwise, the offspring enters the current population, but only under condition that it is better than the worst population member. The worst individual is removed in this case. This our replacement strategy ensures that only individuals that are diverse enough survive for the further generations.

There are a few replacement variations (options), depending on the parameter RepVar. If RepVar=1, then exactly the above replacement strategy is adopted. If RepVar=2, then the new offspring replaces the best member of the current population if the offspring is better than the best population individual. If the offspring is worse than the best individual, then RepVar=2 is identical to RepVar=3. If RepVar=3, then the offspring replaces the worst member of the population, ignoring the fitness of the worst individual. The minimum distance criterion must be taken into account.

### 3.6. Population Restart

The important feature of our genetic algorithm is the use of the population restart mechanism to try to avoid the premature convergence and search stagnation. The restart process is triggered in the situations where the solutions of the population do not change at all for some number of consecutive generations. This can be operationalized by the use of a priori parameter called an idle generations limit, $L_{idle\_gen\;}$, where $L_{idle\_gen}=\max\left\{L_{min},\;\lfloor \omega N_{gen}\rfloor \right\}$, here $L_{min}$ is a constant (we use $L_{min}=3$), ω is to denote a stagnation control parameter and $N_{gen}$ is the total number of generations of the genetic algorithm. (The standard value of ω is equal to 0.05.) The restart itself is performed by applying a so-called multi-mutation, where the mutation process is applied to all the members of the stagnated population. Such approach is preferred to the complete destroying of the population, which seems to be too aggressive.

## 4. Computational Experiments

Our genetic-hierarchical algorithm is implemented by using C# programming language. The computational experiments have been carried out on a 3.1 GHz personal computer running Windows 7 Enterprise. The CPU model is an Intel Core i5-3450.

The algorithm is tested on the small-, medium-and large-scaled QAP benchmark instances of sizes from n=10 to n=128. Most instances are from the online QAP library QAPLIB [29]. Other instances are from [14,19] (see also http:/mistic.heig-vd.ch/taillard/problemes.dir/qap.dir/qap.html).

In particular, the following benchmark instances taken from QAPLIB were examined:

(a) random, unstructured instances (these instances are denoted by: rou20, tai10a, tai12a, tai15a, tai17a, tai20a, tai25a, tai30a, tai35a, tai40a, tai50a, tai60a, tai80a, tai100a);
(b) randomly generated, grid-based instances (they are denoted by: had20, nug30, scr20, sko42, sko49, sko56, sko64, sko72, sko81, sko90, sko100a..sko100f, tho30, tho40, wil50, wil100);
(c) real-life, structured instances from practical applications (denoted by: chr25a, els19, esc32a..esc32h, esc64a, esc128, kra30a, kra30b, ste36a. ste36c, tai64c);
(d) real-life like (pseudo-random), structured instances (denoted by: tai10b, tai12b, tai15b, tai20b, tai25b, tai30b, tai35b, tai40b, tai50b, tai60b, tai80b, tai100b).
(e) instances with known optimal solutions (denoted by: lipa20a, lipa20b, lipa30a, lipa30b, lipa40a, lipa40b, lipa50a, lipa50b, lipa60a, lipa60b, lipa70a, lipa70b, lipa80a, lipa80b, lipa90a, lipa90b).

In addition, the instances introduced by de Carvalho and Rahmann [19] are investigated. These instances are extremely difficult to solve. They are denoted by bl36, bl49, bl64, bl81, bl100 (aka. border length minimization instances) and ci36, ci49, ci64, ci81, ci100 (aka. conflict index minimization instances).

We also tested the instances dre15, dre18, dre21, dre24, dre28, dre30, dre42, dre56, dre72, dre90, tai27e1, tai27e2, tai27e3, tai45e1, tai45e2, tai45e3, tai75e1, tai75e2, tai75e3 proposed by Drezner and Taillard in [14].

In the initial computational experiments, we used the following “learning set” of the QAP benchmark instances of sizes from n=35 to n=70: bl49, bl64, ci49, ci64, dre42, dre56, lipa70a, lipa70b, sko56, sko64, tai35a, tai35b, tai40a, tai40b, tai45e1, tai50a, tai50b, tai60a, tai60b, wil50. These particular instances were chosen based on our preliminary experience.

As a performance criterion, we adopt the average relative percentage deviation ($\bar{\theta }$) of the yielded solutions from the best known solution (BKS). It is calculated by the following formula: $\bar{\theta }=100\frac{\left(\bar{z}-z_{bkv}\right)}{z_{bkv}}\left[\%\right]$, where $\bar{z}$ is the average objective function value over 10 runs of the algorithm, while $z_{bkv}$ denotes the best known value (BKV) of the objective function that corresponds to the BKS (BKVs are from [14,29,86]).

At every run, the algorithm is applied to the particular instance. Each time, the algorithm starts from a new random initial population. The algorithm is terminated if either the maximum number of generations, $N_{gen}$, has been reached or the best known solution has been achieved.

In the experiments, the goal was to empirically test the performance of the basic setup of our algorithm and also its various variants in terms of the quality of solutions and the run time of the algorithm. To do so, we have identified some essential algorithm’s components (ingredients) (namely, “initial population”, “selection”, “crossover”, “local improvement (hierarchical tabu search)”, “mutation”, “population replacement”) to reveal their influence on the efficiency of GHA and to “synthesize” the preferable fine-tuned architecture of the algorithm. The following combination of the particular options (parameters) related to these components is declared as the basic version of GHA: {InitPopVar=1, PS=10, $N_{gen}=100$, σ=1.5, CrossVar=“1PX”, τ=20, $Q_{hier}=2^{8}=256$, MutVar=“M1”, RepVar=1}; here, $Q_{hier}$ denotes the total cumulative number of hierarchical iterations ($Q_{hier}=Q^{\left(0\right)}\times Q^{\left(1\right)}\times Q^{\left(2\right)}\times Q^{\left(3\right)}\times Q^{\left(4\right)}\times Q^{\left(5\right)}\times Q^{\left(6\right)}\times Q^{\left(7\right)}$), $Q^{\left(0\right)},\;\ldots ,\;Q^{\left(7\right)}$ denote, respectively, the corresponding numbers of iterations of the 0th-level, …, 7th-level hierarchical iterated tabu search algorithms. The prescribed default values of the control parameters corresponding to the basic version of GHA are shown in Table 1 and Table 2. (These values were later over-ridden in particular separate experiments).

In the initial experiments, twelve crossover procedures (1PX, 2PX, UX, SX, PMX, SPX1, SPX2, SPX3, CX, COHX, MPX, UNIVX) have been compared against each other. The obtained results (presented in Table 3) demonstrate that our proposed universal crossover (UNIVX) with the tuned control parameters yields the most promising results.

In the further experiments, the different mutation procedures (M1, M2, M3, M4, M5, M6, M7, M8, M9, M10, M11, M12) were examined. This time, we have found out that the randomized tabu search based mutation is clearly the best among the all tested mutation variants (see Table 4).

Further, we were interested in how various options (configurations) of the initial population construction affect the performance of the genetic-hierarchical algorithm. The particular separate configurations differ with respect to the option of the population construction (InitPopVar), the size of pre-initial population ($PS^{\prime }$), as well as the number of TS iterations during the population initialization ($\tau^{\prime }$). In particular, the following variants were investigated: (1) InitPopVar=1, $PS^{\prime }=10$, $\tau^{\prime }=20$; (2) InitPopVar=1, $PS^{\prime }=20$, $\tau^{\prime }=40$; (3) InitPopVar=1, $PS^{\prime }=40$, $\tau^{\prime }=80$; (4) InitPopVar=1, $PS^{\prime }=100$, $\tau^{\prime }=200$; (5) InitPopVar=2, $PS^{\prime }=10$, $\tau^{\prime }=20$; (6) InitPopVar=2, $PS^{\prime }=20$, $\tau^{\prime }=40$; (7) InitPopVar=2, $PS^{\prime }=40$, $\tau^{\prime }=80$; (8) InitPopVar=2, $PS^{\prime }=100$, $\tau^{\prime }=200$; (9) InitPopVar=3, $PS^{\prime }=10$, $\tau^{\prime }=20$; (10) InitPopVar=3, $PS^{\prime }=20$, $\tau^{\prime }=40$; (11) InitPopVar=3, $PS^{\prime }=40$, $\tau^{\prime }=80$; (12) InitPopVar=3, $PS^{\prime }=100$, $\tau^{\prime }=200$.

We have observed that maintaining the higher quality initial populations, in general, allows to significantly increase the overall efficiency of GHA when comparing to the lower quality initial populations (see Table 5).

Additionally, we experimented with some few population replacement options. The particular population replacement variants are as follows: (1) $PS^{\prime }=10$, $\tau^{\prime }=20$, RepVar=1; (2) $PS^{\prime }=10$, $\tau^{\prime }=20$, RepVar=2; (3) $PS^{\prime }=10$, $\tau^{\prime }=20$, RepVar=3; (4) $PS^{\prime }=20$, $\tau^{\prime }=40$, RepVar=1; (5) $PS^{\prime }=20$, $\tau^{\prime }=40$, RepVar=2; (6) $PS^{\prime }=20$, $\tau^{\prime }=40$, RepVar=3; (7) $PS^{\prime }=40$, $\tau^{\prime }=80$, RepVar=1; (8) $PS^{\prime }=40$, $\tau^{\prime }=80$, RepVar=2; (9) $PS^{\prime }=40$, $\tau^{\prime }=80$, RepVar=3; (10) $PS^{\prime }=100$, $\tau^{\prime }=200$, RepVar=1; (11) $PS^{\prime }=100$, $\tau^{\prime }=200$, RepVar=2; (12) $PS^{\prime }=100$, $\tau^{\prime }=200$, RepVar=3.

It was observed that the aggressive strategy of replacement of the best population member (RepVar=2) seems to be superior to other options (see Table 6). Further, more extensive experiments are required to strengthen this conjecture.

In addition, we have tested some other different scenarios (regimes) in order to unveil some possible tendencies of the behaviour of the HITS algorithm. The following scenarios were investigated: (1) scenario of “quick search”: small value of τ—small value of $Q_{hier}$; (2) scenario of “diversified quick search”: small value of τ—large value of $Q_{hier}$; (3) scenario of “extensive search”: large value of τ—small value of $Q_{hier}$; (4) scenario of “diversified extensive search”: large value of τ—large value of $Q_{hier}$. Note that, in these scenarios, the number of generations of GHA should be accordingly balanced in order to stay within the fixed run time. The corresponding values of the control parameters are as follows: (1-a) τ=20, $Q_{hier}=2^{8}=256$, $N_{gen}=100$; (1-b) τ=25, $Q_{hier}=2^{8}=256$, $N_{gen}=80$; (1-c) τ=40, $Q_{hier}=2^{8}=256$, $N_{gen}=50$; (2-a) τ=50, $Q_{hier}=2^{8}=256$, $N_{gen}=40$; (2-b) τ=10, $Q_{hier}=2^{9}=512$, $N_{gen}=100$; (2-c) τ=20, $Q_{hier}=2^{9}=512$, $N_{gen}=50$; (3-a) τ=10, $Q_{hier}=5\times 2^{7}=640$, $N_{gen}=80$; (3-b) τ=20, $Q_{hier}=5\times 2^{7}=640$, $N_{gen}=40$; (3-c) τ=10, $Q_{hier}=2^{10}=1024$, $N_{gen}=50$; (4-a) τ=20, $Q_{hier}=2^{10}=1024$, $N_{gen}=25$; (4-b) τ=10, $Q_{hier}=10\times 2^{7}=1280$, $N_{gen}=40$; (4-c) τ=20, $Q_{hier}=10\times 2^{7}=1280$, $N_{gen}=20$.

The results of the experiments (see Table 7) demonstrate that the scenario of diversified extensive search is obviously preferable to other examined scenarios.

Additional scenarios have been examined to reveal the reaction of GHA when extensively increasing the cumulative number of iterations of the hierarchical iterated tabu search algorithm—$Q_{hier}$. The computational budget is not constant (“balanced”) anymore, but grows as the value of $Q_{hier}$ increases. The following scenarios have been tried: (1) PS=10, $PS^{\prime }=150$, $\tau^{\prime }=300$, $Q_{hier}=5\times 2^{7}=640$; (2) PS=10, $PS^{\prime }=150$, $\tau^{\prime }=300$, $Q_{hier}=6\times 2^{7}=768$; (3) PS=10, $PS^{\prime }=150$, $\tau^{\prime }=300$, $Q_{hier}=7\times 2^{7}=896$; (4) PS=10, $PS^{\prime }=150$, $\tau^{\prime }=300$, $Q_{hier}=8\times 2^{7}=1024$; (5) PS=20, $PS^{\prime }=300$, $\tau^{\prime }=300$, $Q_{hier}=5\times 2^{7}=640$; (6) PS=20, $PS^{\prime }=300$, $\tau^{\prime }=300$, $Q_{hier}=6\times 2^{7}=768$; (7) PS=20, $PS^{\prime }=300$, $\tau^{\prime }=300$, $Q_{hier}=7\times 2^{7}=896$; (8) PS=20, $PS^{\prime }=300$, $\tau^{\prime }=300$, $Q_{hier}=8\times 2^{7}=1024$; (9) PS=10, $PS^{\prime }=200$, $\tau^{\prime }=400$, $Q_{hier}=7\times 2^{7}=896$; (10) PS=10, $PS^{\prime }=200$, $\tau^{\prime }=400$, $Q_{hier}=7\times 2^{7}=1024$; (11) PS=20, $PS^{\prime }=400$, $\tau^{\prime }=400$, $Q_{hier}=7\times 2^{7}=896$; (12) PS=20, $PS^{\prime }=400$, $\tau^{\prime }=400$, $Q_{hier}=7\times 2^{7}=1024$; here, $\tau^{\prime }$ denotes the number of TS iterations during the construction of the initial population.

The results confirm that, as expected, there exists a clear correlation between the number of improving iterations (the number of TS/HITS iterations) and the quality of the obtained solutions (see Table 8).

**Figure A1:**
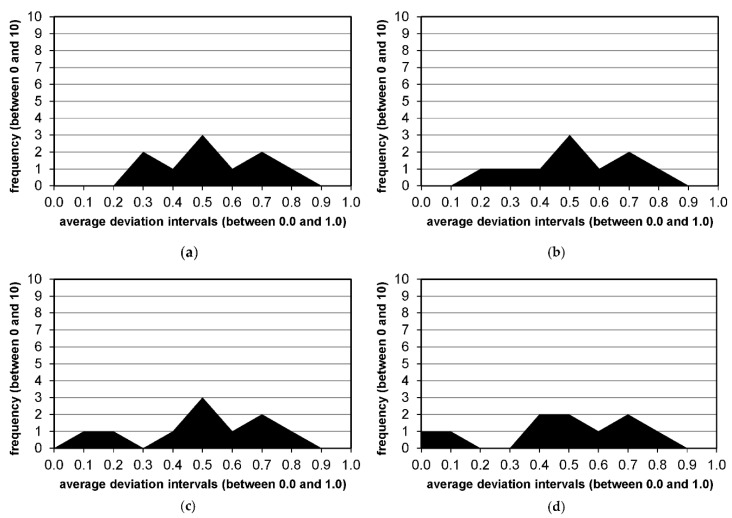
Histograms of the frequency of the objective function values for the instance bl64 for different examined scenarios: (**a**) PS=10, $PS^{\prime }=150$, $\tau^{\prime }=300$, $Q_{hier}=640$; (**b**) PS=10, $PS^{\prime }=150$, $\tau^{\prime }=300$, $Q_{hier}=768$; (**c**) PS=10, $PS^{\prime }=150$, $\tau^{\prime }=300$, $Q_{hier}=896$; (**d**) PS=10, $PS^{\prime }=150$, $\tau^{\prime }=300$, $Q_{hier}=1024$. The histograms are developed in such a way that the frequency of the average deviation is visualized over 10 discrete sub-intervals of the interval [0.0,  : [0.0, 0.1); [0.1, 0.2); [0.2, 0.3); …; [0.9, 1.0). It can be seen that the average deviation from (pseudo-)optimal solutions stably decreases by increasing the number of search iterations ($Q_{hier}$).

To have a reflection of the obtained results from a different perspective—in particular, a demonstration of the stability and robustness properties of our algorithm—we have constructed histograms of the frequency of the objective function values for one of the most difficult instances of the “learning set”—bl64 (see Figure A1 in the “Appendix A” Section). In fact, we have created the histograms of the frequency of the average percentage deviation, $\bar{\theta }$, over 10 algorithm runs within the interval [0.0, 1.0), where 0.0 stands for zero deviation and 1.0 means the maximum possible deviation. (Note that the average deviation never exceeded 1.0 for the instance bl64 (see Table 8).)

(Regarding the selection factor, σ, the obtained results are quite “flat” and not statistically significant, so they are omitted).

On the whole, we have found the best known solutions in the 9191 cases (runs) out of 14400 cases (64% of cases). The BKS was found at least once for all examined instances. The cumulative average percentage deviation is equal to 1.452% and the cumulative average CPU time per run is equal to approximately 65.9 s. The average deviation is less than 0.5 in 73% of cases, while the average deviation is less than 1.0 in 89% of cases. 14 instances (ci49, ci64, dre42, lipa70a, lipa70b, sko56, sko64, tai35a, tai35b, tai40b, tai45e1, tai50b, tai60b, wil50) were solved to pseudo-optimality in more than 300 runs.

After experimenting with the “learning set” of instances, the other instances (the “testing set” of instances) were examined using the fine-tuned parameters in order to find out how quickly the genetic-hierarchical algorithm converges to the best known/optimal solutions. The obtained results are presented in Table 9. It can be seen that all tested instances (88 instances) are solved to pseudo-optimality within extremely small computation time.

We have also compared our algorithm with the memetic algorithm (MA) proposed be Benlic and Hao [78], which is most likely the best so far heuristic algorithm for the QAP, to the best of our knowledge. The results of comparison of the algorithms are presented in Table 10, Table 11 and Table 12. It seems that our genetic-hierarchical algorithm outperforms MA. Additionally, we used the genetic algorithms by Drezner et al. [14] and Drezner and Misevičius [86] in the further comparison (see Table 13, Table 14, Table 15 and Table 16). Again, our algorithm compares favourably to both the algorithm by Drezner et al. as well as Drezner and Misevičius.

## 5. Concluding Remarks

In this paper, we have presented the hybrid genetic-hierarchical algorithm for the solution of the quadratic assignment problem. The key feature of the proposed algorithm is that the genetic algorithm is hybridized with the hierarchicity-based (self-similar) iterated tabu search algorithm, which serves as a powerful local optimizer of the offspring solutions produced by the crossover operator.

The algorithm was examined on the QAP benchmark instances of various sizes and complexity. The results obtained from the experiments demonstrate the excellent performance of the genetic-hierarchical algorithm. Our algorithm seems to outperform other state-of-the-art heuristic algorithms for many examined QAP instances or is at least very much competitive with them. A more pronounced improvement in the quality of the results might be achieved by a thorough calibration of the algorithm’s parameters.

The following are some possible future research directions: balancing of the number of tabu search iterations and the number of hierarchical iterated tabu search iterations, as well as the number of hierarchical levels; extensive experimental analysis of the particular components and configurations of the genetic-hierarchical algorithm; designing and implementing a multi-level hierarchical (master-slave) genetic algorithm.

## Figures and Tables

**Table 1:** Values of the control parameters of the basic version of GHA used in the experiments.

Parameter	Value	Remarks
Population size, PS	10	
Number of generations, $N_{gen}$	100	
Initial population variant, InitPopVar	1	
Selection factor, σ	1.5	
Distance threshold, DT	max{2, ⌊0.05n⌋}	0<DT≤n
Idle generations limit, $L_{idle\_gen}$	$\max\left\{3,\;\lfloor 0.05N_{gen}\rfloor \right\}$	$0<L_{idle\_gen}\leq N_{gen}$
Population replacement variant, RepVar	1	

**Table 2:** Standard values of the control parameters of the hierarchical iterated tabu search algorithm.

Parameter	Value	Remarks
Number of hierarchical iterated tabu search iterations, $Q_{hier}$	256	$Q_{hier}=Q^{\left(0\right)}\times Q^{\left(1\right)}\times Q^{\left(2\right)}\times Q^{\left(3\right)}\times$ $\;\;\;\;\;\;\;\;\;\;\;\;\;\;\;\;Q^{\left(4\right)}\times Q^{\left(5\right)}\times Q^{\left(6\right)}\times Q^{\left(7\right)}$ ^†^
Number of tabu search iterations, τ	20	
Idle iterations limit, $L_{idle\_iter}$	⌊0.2τ⌋	$0<L_{idle\_iter}\leq \tau$
Tabu tenure, h	⌊0.3n⌋	h>0
Randomization coefficient for tabu search, α	0.02	0≤α<1
Mutation rate, ξ	⌊0.2n⌋	2≤ξ≤n

^†^$Q^{\left(0\right)}=Q^{\left(1\right)}=Q^{\left(2\right)}=Q^{\left(3\right)}=Q^{\left(4\right)}=Q^{\left(5\right)}=Q^{\left(6\right)}=Q^{\left(7\right)}=2$.

**Table 3:** Comparison of crossover procedures.

Instance	BKV	$\bar{\theta }$												Time (s)
		1PX	2PX	UX	SX	PMX	SPX1	SPX2	SPX3	CX	COHX	MPX	UNIVX	
**bl49**	4548	0.730	0.765	0.712	0.756	0.756	0.812	0.792	0.730	0.765	0.712	0.853	0.688	*63.9*
**bl64**	5988	1.009	0.775	0.768	1.222	1.062	0.795	1.136	1.096	0.882	0.962	1.416	0.962	*126.7*
**ci49**	236355034	0.004	0.002	0.009	0.000	0.012	0.004	0.000	0.000	0.003	0.013	0.003	0.005	*10.7*
**ci64**	325671035	0.092	0.086	0.103	0.087	0.081	0.097	0.066	0.055	0.085	0.068	0.110	0.098	*65.4*
**dre42**	764	8.770	15.052	13.665	11.990	11.126	18.770	9.738	8.586	14.058	14.607	7.696	7.408	*18.1*
**dre56**	1086	28.785	28.674	36.206	37.661	35.470	39.282	38.122	38.471	29.963	24.199	46.335	26.298	*59.6*
**lipa70a**	169755	0.165	0.165	0.165	0.165	0.165	0.165	0.165	0.165	0.165	0.165	0.165	0.165	*23.3*
**lipa70b**	4603200	0.000	0.000	0.000	0.000	0.000	0.000	0.000	0.000	0.000	0.000	0.000	0.000	*1.8*
**sko56**	34458	0.002	0.001	0.018	0.000	0.017	0.021	0.002	0.000	0.002	0.000	0.014	0.001	*16.9*
**sko64**	48498	0.006	0.000	0.022	0.000	0.000	0.016	0.000	0.000	0.000	0.000	0.015	0.006	*8.4*
**tai35a**	2422002	0.355	0.416	0.332	0.263	0.233	0.506	0.313	0.239	0.386	0.252	0.215	0.484	*16.0*
**tai35b**	283315445	0.000	0.000	0.000	0.000	0.000	0.019	0.000	0.000	0.000	0.000	0.000	0.000	*1.5*
**tai40a**	3139370	0.483	0.417	0.556	0.477	0.482	0.686	0.464	0.462	0.513	0.622	0.534	0.601	*39.1*
**tai40b**	637250948	0.000	0.000	0.000	0.000	0.000	0.000	0.000	0.000	0.000	0.000	0.000	0.000	*2.0*
**tai45e1**	6412	3.253	1.366	2.944	1.307	1.285	2.682	0.172	1.597	3.506	2.327	6.323	1.482	*29.2*
**tai50a**	4938796	0.810	0.834	0.872	0.742	0.712	0.960	0.784	0.884	0.829	0.797	0.838	0.912	*67.1*
**tai50b**	458821517	0.000	0.033	0.033	0.000	0.000	0.000	0.000	0.033	0.033	0.066	0.113	0.033	*3.9*
**tai60a**	7205962	0.819	0.826	0.904	0.894	0.858	0.976	0.899	0.865	0.971	0.762	0.901	0.888	*103.4*
**tai60b**	608215054	0.037	0.000	0.000	0.000	0.037	0.000	0.000	0.000	0.000	0.000	0.040	0.000	*6.8*
**wil50**	48816	0.002	0.003	0.013	0.007	0.007	0.011	0.008	0.000	0.005	0.007	0.007	0.011	*6.2*
	**Average:**	**2.266**	**2.471**	**2.866**	**2.779**	**2.615**	**3.290**	**2.633**	**2.659**	**2.608**	**2.278**	**3.279**	**2.001**	

Notes. Time denotes the average CPU time per one run. In all cases, the first mutation variant (M1) is used.

**Table 4:** Comparison of mutation procedures.

Instance	BKV	$\bar{\theta }$												Time (s)
		M1	M2	M3	M4	M5	M6	M7	M8	M9	M10	M11	M12	
**bl49**	4548	0.730	0.800	0.994	0.712	0.739	0.642	1.099	0.976	1.020	1.082	0.624	0.792	*63.8*
**bl64**	5988	1.009	1.075	1.336	0.855	0.862	1.049	1.229	1.376	1.229	2.057	0.755	1.336	*125.2*
**ci49**	236355034	0.004	0.004	0.080	0.021	0.000	0.001	0.097	0.003	0.003	0.000	0.001	0.001	*9.6*
**ci64**	325671035	0.092	0.085	0.218	0.089	0.074	0.092	0.187	0.256	0.110	0.083	0.075	0.049	*66.3*
**dre42**	764	8.770	11.204	22.984	1.466	15.026	7.016	25.916	20.524	14.346	16.335	8.063	0.000	*18.9*
**dre56**	1086	28.785	32.431	40.829	26.538	29.705	37.459	41.197	39.576	34.494	56.814	26.206	16.777	*58.1*
**lipa70a**	169755	0.165	0.165	0.165	0.165	0.165	0.165	0.165	0.165	0.165	0.165	0.165	0.165	*24.0*
**lipa70b**	4603200	0.000	0.000	0.000	0.000	0.000	0.000	0.000	0.000	0.000	0.000	0.000	0.000	*2.1*
**sko56**	34458	0.002	0.000	0.082	0.019	0.000	0.000	0.096	0.064	0.003	0.001	0.000	0.000	*16.4*
**sko64**	48498	0.006	0.000	0.002	0.006	0.000	0.000	0.007	0.026	0.001	0.000	0.006	0.000	*8.3*
**tai35a**	2422002	0.355	0.386	0.707	0.377	0.240	0.365	0.672	0.520	0.513	0.000	0.197	0.034	*17.2*
**tai35b**	283315445	0.000	0.000	0.000	0.084	0.000	0.000	0.000	0.000	0.037	0.000	0.000	0.028	*1.3*
**tai40a**	3139370	0.483	0.501	0.797	0.508	0.487	0.520	0.771	0.652	0.699	0.337	0.448	0.289	*40.1*
**tai40b**	637250948	0.000	0.201	0.000	0.000	0.000	0.000	0.000	0.201	0.402	0.000	0.000	0.603	*2.2*
**tai45e1**	6412	3.253	1.376	10.231	11.784	2.714	2.246	9.454	8.456	17.034	0.000	1.301	15.490	*30.5*
**tai50a**	4938796	0.810	0.718	1.193	0.876	0.813	0.846	1.064	1.044	0.899	0.601	0.737	0.487	*67.5*
**tai50b**	458821517	0.000	0.000	0.078	0.253	0.000	0.033	0.019	0.033	0.035	0.000	0.033	0.123	*3.7*
**tai60a**	7205962	0.819	0.879	1.123	0.908	0.883	0.882	1.200	1.041	0.935	0.649	0.830	0.487	*103.7*
**tai60b**	608215054	0.037	0.000	0.002	0.201	0.000	0.000	0.037	0.005	0.000	0.000	0.000	0.409	*6.9*
**wil50**	48816	0.002	0.005	0.017	0.018	0.003	0.003	0.025	0.011	0.014	0.000	0.007	0.000	*6.6*
	**Average:**	**2.266**	**2.492**	**4.042**	**2.244**	**2.586**	**3.290**	**4.162**	**3.746**	**3.597**	**3.906**	**1.972**	**1.854**	

Note. In all cases, the 1PX crossover is used.

**Table 5:** Comparison of different variants of the initial population construction.

Instance	BKV	$\bar{\theta }$												Time (s)
		(1)	(2)	(3)	(4)	(5)	(6)	(7)	(8)	(9)	(10)	(11)	(12)	
**bl49**	4548	0.607	0.589	0.554	0.607	0.668	0.598	0.589	0.624	0.616	0.598	0.519	0.493	*115.2*
**bl64**	5988	0.601	0.835	0.741	0.661	0.741	0.501	0.768	0.681	0.735	0.681	0.715	0.661	*243.1*
**ci49**	236355034	0.000	0.000	0.000	0.000	0.000	0.000	0.000	0.000	0.000	0.000	0.000	0.000	*16.3*
**ci64**	325671035	0.055	0.019	0.029	0.000	0.051	0.060	0.028	0.000	0.008	0.007	0.000	0.000	*81.6*
**dre42**	764	4.267	3.272	6.466	0.000	6.309	4.869	4.293	0.000	1.335	0.000	0.000	0.000	*16.7*
**dre56**	1086	23.462	20.626	12.302	4.494	13.131	22.118	13.941	11.013	20.166	19.153	4.807	3.757	*129.8*
**lipa70a**	169755	0.055	0.055	0.055	0.055	0.055	0.055	0.055	0.055	0.055	0.055	0.055	0.055	*18.8*
**lipa70b**	4603200	0.000	0.000	0.000	0.000	0.000	0.000	0.000	0.000	0.000	0.000	0.000	0.000	*7.8*
**sko56**	34458	0.000	0.000	0.000	0.000	0.000	0.000	0.000	0.000	0.000	0.000	0.000	0.000	*9.9*
**sko64**	48498	0.000	0.000	0.000	0.000	0.000	0.000	0.000	0.000	0.000	0.000	0.000	0.000	*11.5*
**tai35a**	2422002	0.201	0.169	0.076	0.000	0.127	0.081	0.000	0.000	0.000	0.000	0.000	0.000	*22.0*
**tai35b**	283315445	0.000	0.000	0.000	0.000	0.000	0.000	0.000	0.000	0.000	0.000	0.000	0.000	*9.4*
**tai40a**	3139370	0.443	0.377	0.335	0.219	0.512	0.277	0.263	0.083	0.311	0.231	0.088	0.067	*66.8*
**tai40b**	637250948	0.000	0.000	0.000	0.000	0.000	0.000	0.000	0.000	0.000	0.000	0.000	0.000	*2.2*
**tai45e1**	6412	0.730	0.730	0.000	0.000	0.000	0.000	0.000	0.000	0.000	0.000	0.000	0.000	*36.6*
**tai50a**	4938796	0.647	0.715	0.628	0.450	0.620	0.610	0.560	0.352	0.577	0.488	0.372	0.191	*120.4*
**tai50b**	458821517	0.000	0.000	0.000	0.000	0.000	0.000	0.000	0.000	0.000	0.000	0.000	0.000	*4.7*
**tai60a**	7205962	0.721	0.672	0.643	0.519	0.667	0.660	0.549	0.463	0.633	0.506	0.460	0.353	*194.9*
**tai60b**	608215054	0.037	0.000	0.000	0.000	0.000	0.000	0.000	0.000	0.000	0.000	0.000	0.000	*60.1*
**wil50**	48816	0.003	0.000	0.000	0.000	0.000	0.000	0.000	0.000	0.000	0.000	0.000	0.000	*5.4*
	**Average:**	**1.591**	**1.403**	**1.091**	**0.350**	**1.144**	**1.491**	**1.052**	**0.664**	**1.222**	**1.086**	**0.351**	**0.279**	

Note. In all cases, the UNIVX crossover and the twelfth mutation variant (M12) are used.

**Table 6:** Comparison of different variants of population replacement.

Instance	BKV	$\bar{\theta }$												Time (s)
		(1)	(2)	(3)	(4)	(5)	(6)	(7)	(8)	(9)	(10)	(11)	(12)	
**bl49**	4548	0.607	0.624	0.677	0.589	0.642	0.580	0.554	0.624	0.616	0.607	0.589	0.589	*62.1*
**bl64**	5988	0.601	0.635	0.715	0.835	0.715	0.688	0.741	0.695	0.768	0.661	0.635	0.755	*123.0*
**ci49**	236355034	0.000	0.000	0.000	0.000	0.000	0.000	0.000	0.000	0.000	0.000	0.000	0.000	*10.2*
**ci64**	325671035	0.055	0.035	0.040	0.019	0.051	0.036	0.029	0.032	0.027	0.000	0.000	0.000	*67.7*
**dre42**	764	4.267	6.963	8.246	3.272	1.492	2.094	6.466	1.466	5.419	0.000	0.000	0.000	*19.6*
**dre56**	1086	23.462	15.488	9.687	20.626	11.326	18.250	12.302	8.122	10.055	4.494	5.783	7.035	*57.0*
**lipa70a**	169755	0.055	0.055	0.055	0.055	0.055	0.055	0.055	0.055	0.055	0.055	0.055	0.055	*24.3*
**lipa70b**	4603200	0.000	0.000	0.000	0.000	0.000	0.000	0.000	0.000	0.000	0.000	0.000	0.000	*3.1*
**sko56**	34458	0.000	0.001	0.000	0.000	0.000	0.000	0.000	0.000	0.000	0.000	0.000	0.000	*17.2*
**sko64**	48498	0.000	0.000	0.000	0.000	0.000	0.000	0.000	0.000	0.000	0.000	0.000	0.000	*7.7*
**tai35a**	2422002	0.201	0.222	0.142	0.169	0.130	0.165	0.076	0.076	0.032	0.000	0.000	0.000	*17.8*
**tai35b**	283315445	0.000	0.000	0.000	0.000	0.000	0.000	0.000	0.000	0.000	0.000	0.000	0.000	*2.1*
**tai40a**	3139370	0.443	0.410	0.444	0.377	0.415	0.438	0.335	0.326	0.296	0.219	0.219	0.222	*38.5*
**tai40b**	637250948	0.000	0.000	0.000	0.000	0.000	0.000	0.000	0.000	0.000	0.000	0.000	0.000	*1.9*
**tai45e1**	6412	0.730	2.920	2.492	0.730	1.023	0.605	0.000	0.000	0.459	0.000	0.000	0.000	*33.2*
**tai50a**	4938796	0.647	0.701	0.648	0.715	0.671	0.685	0.628	0.611	0.606	0.450	0.450	0.424	*66.7*
**tai50b**	458821517	0.000	0.000	0.000	0.000	0.000	0.000	0.000	0.000	0.000	0.000	0.000	0.000	*1.2*
**tai60a**	7205962	0.721	0.740	0.687	0.672	0.690	0.744	0.643	0.627	0.627	0.519	0.469	0.501	*105.9*
**tai60b**	608215054	0.037	0.000	0.000	0.000	0.000	0.000	0.000	0.000	0.000	0.000	0.000	0.000	*6.1*
**wil50**	48816	0.003	0.002	0.003	0.000	0.000	0.000	0.000	0.000	0.000	0.000	0.000	0.000	*6.7*
	**Average:**	**1.591**	**1.440**	**1.192**	**1.403**	**0.861**	**1.217**	**1.091**	**0.632**	**0.948**	**0.350**	**0.410**	**0.479**	

Note. In all cases, the UNIVX crossover and the mutation variant M12 are used. Also, the initial population construction option InitPopVar=1 is used.

**Table 7:** Comparison of different variants of the hierarchical iterated tabu search algorithm.

Instance	BKV	$\bar{\theta }$												Time (s)
		(1-a)	(1-b)	(1-c)	(2-a)	(2-b)	(2-c)	(3-a)	(3-b)	(3-c)	(4-a)	(4-b)	(4-c)	
**bl49**	4548	0.598	0.545	0.633	0.633	0.440	0.589	0.528	0.475	0.528	0.510	0.475	0.510	*125.1*
**bl64**	5988	0.735	0.675	0.788	0.935	0.721	0.715	0.661	0.808	0.695	0.681	0.675	0.755	*350.5*
**ci49**	236355034	0.000	0.000	0.000	0.000	0.000	0.000	0.000	0.000	0.000	0.000	0.000	0.000	*15.3*
**ci64**	325671035	0.031	0.014	0.019	0.021	0.011	0.017	0.004	0.011	0.007	0.000	0.004	0.000	*65.1*
**dre42**	764	3.613	1.466	0.000	2.670	0.000	1.335	0.000	0.000	0.000	0.000	0.000	0.000	*27.2*
**dre56**	1086	8.692	13.297	14.512	16.133	17.017	19.227	13.223	7.974	9.816	5.506	6.851	5.672	*180.7*
**lipa70a**	169755	0.052	0.000	0.000	0.000	0.000	0.000	0.000	0.000	0.000	0.000	0.000	0.000	*23.4*
**lipa70b**	4603200	0.000	0.000	0.000	0.000	0.000	0.000	0.000	0.000	0.000	0.000	0.000	0.000	*18.4*
**sko56**	34458	0.000	0.000	0.000	0.000	0.000	0.000	0.000	0.000	0.000	0.000	0.000	0.000	*29.3*
**sko64**	48498	0.000	0.000	0.000	0.000	0.000	0.000	0.000	0.000	0.000	0.000	0.000	0.000	*28.4*
**tai35a**	2422002	0.108	0.136	0.000	0.000	0.077	0.020	0.063	0.078	0.000	0.000	0.067	0.000	*37.0*
**tai35b**	283315445	0.000	0.000	0.000	0.000	0.000	0.000	0.000	0.000	0.000	0.000	0.000	0.000	*19.8*
**tai40a**	3139370	0.342	0.337	0.350	0.277	0.362	0.264	0.315	0.275	0.322	0.263	0.267	0.201	*132.7*
**tai40b**	637250948	0.000	0.000	0.000	0.000	0.000	0.000	0.000	0.000	0.000	0.000	0.000	0.000	*11.9*
**tai45e1**	6412	0.586	0.730	0.293	2.015	0.000	0.293	0.000	0.000	0.000	0.000	0.000	0.000	*67.7*
**tai50a**	4938796	0.487	0.599	0.559	0.499	0.613	0.612	0.614	0.527	0.504	0.524	0.481	0.491	*281.1*
**tai50b**	458821517	0.000	0.000	0.000	0.000	0.000	0.000	0.000	0.000	0.000	0.000	0.000	0.000	*33.3*
**tai60a**	7205962	0.649	0.607	0.620	0.582	0.573	0.564	0.562	0.635	0.543	0.584	0.542	0.511	*345.5*
**tai60b**	608215054	0.000	0.000	0.000	0.000	0.000	0.000	0.000	0.000	0.000	0.000	0.000	0.000	*36.8*
**wil50**	48816	0.000	0.000	0.000	0.000	0.000	0.000	0.000	0.000	0.000	0.000	0.000	0.000	*8.9*
	**Average:**	**0.795**	**0.920**	**0.889**	**1.188**	**0.991**	**1.182**	**0.799**	**0.539**	**0.621**	**0.403**	**0.468**	**0.407**	

Note. In all cases, the UNIVX crossover and the mutation variant M12 are used. Also, the initial population construction option InitPopVar=1 and population replacement option RepVar=2 are used.

**Table 8:** Results of the experiments with the increased number of iterations of the hierarchical iterated tabu search algorithm.

Instance	BKV	$\bar{\theta }$												Time (s)
		(1)	(2)	(3)	(4)	(5)	(6)	(7)	(8)	(9)	(10)	(11)	(12)	
**bl49**	4548	0.440	0.466	0.466	0.396	0.475	0.484	0.396	0.440	0.484	0.396	0.484	0.466	*230.5*
**bl64**	5988	0.615	0.608	0.568	0.541	0.548	0.528	0.574	0.635	0.508	0.521	0.648	0.581	*650.0*
**ci49**	236355034	0.000	0.000	0.000	0.000	0.000	0.000	0.000	0.000	0.000	0.000	0.000	0.000	*17.3*
**ci64**	325671035	0.000	0.000	0.000	0.000	0.000	0.000	0.000	0.000	0.000	0.000	0.000	0.000	*66.8*
**dre42**	764	0.000	0.000	0.000	0.000	0.000	0.000	0.000	0.000	0.000	0.000	0.000	0.000	*28.4*
**dre56**	1086	5.654	1.344	0.000	1.731	4.696	1.289	0.000	4.678	0.000	2.910	0.000	2.910	*520.0*
**lipa70a**	169755	0.000	0.000	0.000	0.000	0.000	0.000	0.000	0.000	0.000	0.000	0.000	0.000	*23.7*
**lipa70b**	4603200	0.000	0.000	0.000	0.000	0.000	0.000	0.000	0.000	0.000	0.000	0.000	0.000	*19.3*
**sko56**	34458	0.000	0.000	0.000	0.000	0.000	0.000	0.000	0.000	0.000	0.000	0.000	0.000	*29.8*
**sko64**	48498	0.000	0.000	0.000	0.000	0.000	0.000	0.000	0.000	0.000	0.000	0.000	0.000	*29.6*
**tai35a**	2422002	0.000	0.000	0.000	0.000	0.000	0.000	0.000	0.000	0.000	0.000	0.000	0.000	*40.2*
**tai35b**	283315445	0.000	0.000	0.000	0.000	0.000	0.000	0.000	0.000	0.000	0.000	0.000	0.000	*19.3*
**tai40a**	3139370	0.015	0.030	0.037	0.037	0.022	0.037	0.030	0.037	0.037	0.037	0.022	0.022	*199.5*
**tai40b**	637250948	0.000	0.000	0.000	0.000	0.000	0.000	0.000	0.000	0.000	0.000	0.000	0.000	*12.9*
**tai45e1**	6412	0.000	0.000	0.000	0.000	0.000	0.000	0.000	0.000	0.000	0.000	0.000	0.000	*68.8*
**tai50a**	4938796	0.084	0.116	0.117	0.080	0.092	0.152	0.119	0.057	0.036	0.092	0.052	0.011	*291.0*
**tai50b**	458821517	0.000	0.000	0.000	0.000	0.000	0.000	0.000	0.000	0.000	0.000	0.000	0.000	*34.1*
**tai60a**	7205962	0.221	0.258	0.237	0.238	0.235	0.236	0.277	0.221	0.214	0.211	0.200	0.161	*554.0*
**tai60b**	608215054	0.000	0.000	0.000	0.000	0.000	0.000	0.000	0.000	0.000	0.000	0.000	0.000	*37.5*
**wil50**	48816	0.000	0.000	0.000	0.000	0.000	0.000	0.000	0.000	0.000	0.000	0.000	0.000	*10.1*
	**Average:**	**0.351**	**0.141**	**0.071**	**0.151**	**0.303**	**0.136**	**0.070**	**0.303**	**0.064**	**0.208**	**0.070**	**0.208**	

Notes. In all cases, the UNIVX crossover and the mutation variant M12 are used. Also, the options InitPopVar=3, RepVar=2 are used.

**Table 9:** Results of GHA for the set of 88 instances of QAPLIB [14,19,29].

Instance	BKV	$\bar{\theta }$	Time (s)	Instance	BKV	$\bar{\theta }$	Time (s)
**bl36**	3296	0.000	9.491	**lipa90b**	12490441	0.000	0.803
**chr25a**	3796	0.000	1.936	**nug30**	6124	0.000	0.122
**ci36**	168611971	0.000	1.279	**rou20**	725522	0.000	0.089
**ci49**	236355034	0.000	6.864	**scr20**	110030	0.000	0.022
**ci64**	325671035	0.000	46.818	**sko42**	15812	0.000	0.592
**ci81**	427447820	0.000	250.470	**sko49**	23386	0.000	7.989
**dre15**	306	0.000	0.003	**sko56**	34458	0.000	7.145
**dre18**	332	0.000	0.034	**sko64**	48498	0.000	7.833
**dre21**	356	0.000	0.033	**ste36a**	9526	0.000	0.830
**dre24**	396	0.000	0.178	**ste36b**	15852	0.000	0.175
**dre28**	476	0.000	0.470	**ste36c**	8239110	0.000	0.513
**dre30**	508	0.000	0.875	**tai10a**	135028	0.000	0.005
**dre42**	764	0.000	9.809	**tai10b**	1183760	0.000	0.003
**dre56**	1086	0.000	86.024	**tai12a**	224416	0.000	0.003
**dre72**	1452	0.000	489.877	**tai12b**	39464925	0.000	0.003
**els19**	17212548	0.000	0.023	**tai15a**	388214	0.000	0.006
**esc32a**	130	0.000	0.237	**tai15b**	51765268	0.000	0.005
**esc32b**	168	0.000	0.022	**tai17a**	491812	0.000	0.009
**esc32c**	642	0.000	0.005	**tai20a**	703482	0.000	0.122
**esc32d**	200	0.000	0.008	**tai20b**	122455319	0.000	0.014
**esc32e**	2	0.000	0.003	**tai25a**	1167256	0.000	0.262
**esc32f**	2	0.000	0.005	**tai25b**	344355646	0.000	0.041
**esc32g**	6	0.000	0.003	**tai27e1**	2558	0.000	0.332
**esc32h**	438	0.000	0.008	**tai27e2**	2850	0.000	0.399
**esc64a**	116	0.000	0.026	**tai27e3**	3258	0.000	0.078
**esc128**	64	0.000	0.335	**tai30a**	1818146	0.000	0.392
**had20**	6922	0.000	0.013	**tai30b**	637117113	0.000	0.176
**kra30a**	88900	0.000	0.304	**tai35a**	2422002	0.000	1.527
**kra30b**	91420	0.000	0.643	**tai35b**	283315445	0.000	0.800
**lipa20a**	3683	0.000	0.009	**tai40b**	637250948	0.000	0.900
**lipa20b**	27076	0.000	0.002	**tai45e1**	6412	0.000	1.346
**lipa30a**	13178	0.000	0.038	**tai45e2**	5734	0.000	5.713
**lipa30b**	151426	0.000	0.008	**tai45e3**	7438	0.000	2.471
**lipa40a**	31538	0.000	0.190	**tai50b**	458821517	0.000	5.488
**lipa40b**	476581	0.000	0.017	**tai60b**	608215054	0.000	5.036
**lipa50a**	62093	0.000	0.473	**tai64c**	1855928	0.000	0.022
**lipa50b**	1210244	0.000	0.062	**tai75e1**	14488	0.000	52.287
**lipa60a**	107218	0.000	4.446	**tai75e2**	14444	0.000	25.134
**lipa60b**	2520135	0.000	0.153	**tai75e3**	14154	0.000	36.677
**lipa70a**	169755	0.000	6.915	**tai80b**	818415043	0.000	29.161
**lipa70b**	4603200	0.000	0.251	**tai100b**	1185996137	0.000	83.515
**lipa80a**	253195	0.000	22.615	**tho30**	149936	0.000	0.097
**lipa80b**	7763962	0.000	0.579	**tho40**	240516	0.000	3.928
**lipa90a**	360630	0.000	81.371	**wil50**	48816	0.000	3.133

Note. Time denotes the average CPU time per one run.

**Table 10:** Comparative results between GHA and memetic algorithm (MA) [78] (part I).

Instance	BKV	GHA		MA	
		$\bar{\theta }$	Time (s)	$\bar{\theta }$	Time (s)
**sko72**	66256	**0.000**	**29.380**	**0.000**	240.000
**sko81**	90998	**0.000**	**95.421**	**0.000**	258.000
**sko90**	115534	**0.000**	**229.456**	**0.000**	918.000
**sko100a**	152002	**0.000**	**542.640**	**0.000**	1338.000
**sko100b**	153890	**0.000**	**227.774**	**0.000**	390.000
**sko100c**	147862	**0.000**	**400.697**	**0.000**	720.000
**sko100d**	149576	**0.000**	**377.108**	**0.006**	1254.000
**sko100e**	149150	**0.000**	**438.632**	**0.000**	714.000
**sko100f**	149036	**0.000**	**790.550**	**0.000**	1380.000
**wil100**	273038	**0.000**	**600.566**	**0.000**	870.000

Note. Time denotes the average CPU time per one run.

**Table 11:** Comparative results between GHA and memetic algorithm (MA) [78] (part II).

Instance	BKV	GHA		MA	
		$\bar{\theta }$	Time (s)	$\bar{\theta }$	Time (s)
**tai40a**	3139370	**0.052**(**3**)	**204.916**	0.059(2)	486.000
**tai50a**	4938796	0.192(**2**)	**268.705**	**0.131**(**2**)	2520.000
**tai60a**	7205962	0.215(1)	**713.455**	**0.144**(**2**)	4050.000
**tai80a**	13499184	**0.367**(0)	**3040.000**	0.426(0)	3948.000
**tai100a**	21043560	**0.311**(0)	6200.000	0.447(0)	**2646.000**

Notes. Time denotes the average CPU time per one run. In parentheses, we present the number of times that the BKS has been found. The best known value for tai100a is from [140].

**Table 12:** Comparative results between GHA and memetic algorithm (MA) [78] (part III).

Instance	BKV	GHA		MA	
		$\bar{\theta }$	Time (s)	$\bar{\theta }$	Time (s)
**tai50b**	458821517	**0.000**	**5.488**	**0.000**	72.000
**tai60b**	608215054	**0.000**	**5.036**	**0.000**	312.000
**tai80b**	818415043	**0.000**	**29.161**	**0.000**	1878.000
**tai100b**	1185996137	**0.000**	**83.515**	**0.000**	816.000

Note. Time denotes the average CPU time per one run.

**Table 13:** Comparative results between GHA and hybrid genetic algorithm (HGA) [14] (part I).

Instance	BKV	GHA		HGA	
		$\bar{\theta }$	Time (s)	$\bar{\theta }$	Time (s)
**dre30**	508	**0.000**(10)	**0.875**	**0.000**	143.400
**dre42**	764	**0.000**(10)	**9.809**	1.340	547.800
**dre56**	1086	**0.000**(10)	**86.024**	17.460	1810.800
**dre72**	1452	**0.000**(10)	**489.877**	27.280	5591.400
**dre90**	1838	**10.351**(2)	**9999.978**	33.880	11,557.800

Note. Time denotes the average CPU time per one run. In parentheses, we present the number of times that the BKS has been found.

**Table 14:** Comparative results between GHA and hybrid genetic algorithm (HGA) [14] (part II).

Instance	BKV	GHA		HGA	
		$\bar{\theta }$	Time (s)	$\bar{\theta }$	Time (s)
**tai27e1**	2558	**0.000**	**0.332**	**0.000**	~60.000
**tai27e2**	2850	**0.000**	**0.399**	**0.000**	~60.000
**tai27e3**	3258	**0.000**	**0.078**	**0.000**	~60.000
**tai45e1**	6412	**0.000**	**1.346**	**0.000**	~300.000
**tai45e2**	5734	**0.000**	**5.713**	**0.000**	~300.000
**tai45e3**	7438	**0.000**	**2.471**	**0.000**	~300.000
**tai75e1**	14488	**0.000**	**52.287**	**0.000**	~2220.000
**tai75e2**	14444	**0.000**	**25.134**	0.339	~2220.000
**tai75e3**	14154	**0.000**	**36.677**	**0.000**	~2220.000

Note. Time denotes the average CPU time per one run.

**Table 15:** Comparative results between GHA and hybrid genetic algorithm with differential improvement (HGA-DI) [86] (part I).

Instance	BKV	GHA		HGA-DI	
		$\bar{\theta }$	Time (s)	$\bar{\theta }$	Time (s)
**bl36**	3296	**0.000**(**10**)	**9.491**	**0.000**(**10**)	51.000
**bl49**	4548	**0.229**(**2**)	217.540	0.334( 0)	**125.000**
**bl64**	5988	0.294(**1**)	550.060	**0.227**( 0)	**356.000**
**bl81**	7532	**0.490**(0)	1725.800	0.494( 0)	**937.000**
**bl100**	9264	**0.527**(0)	4070.800	0.548( 0)	**2306.000**

Note. Time denotes the average CPU time per one run. In parentheses, we present the number of times that the BKS has been found.

**Table 16:** Comparative results between GHA and hybrid genetic algorithm with differential improvement (HGA-DI) [86] (part II).

Instance	BKV	GHA		HGA-DI	
		$\bar{\theta }$	Time (s)	$\bar{\theta }$	Time (s)
**ci36**	168611971	**0.000**(**10**)	**1.279**	**0.000**(**10**)	50.000
**ci49**	236355034	**0.000**(**10**)	**6.864**	**0.000**(**10**)	124.000
**ci64**	325671035	**0.000**(**10**)	**46.818**	**0.000**(**10**)	354.000
**ci81**	427447820	**0.000**(**10**)	**250.470**	**0.000**(**10**)	932.000
**ci100**	523146366	**0.003**(**7**)	4270.300	0.007( 3)	**2285.000**

Note. Time denotes the average CPU time per one run. In parentheses, we present the number of times that the BKS has been found.

## Data Availability

Not applicable.
